# Attacking the mosquito on multiple fronts: Insights from the Vector Control Optimization Model (VCOM) for malaria elimination

**DOI:** 10.1371/journal.pone.0187680

**Published:** 2017-12-01

**Authors:** Samson S. Kiware, Nakul Chitnis, Allison Tatarsky, Sean Wu, Héctor Manuel Sánchez Castellanos, Roly Gosling, David Smith, John M. Marshall

**Affiliations:** 1 Environmental Health and Ecological Sciences Department, Ifakara Health Institute, Morogoro, Tanzania; 2 Mathematics, Statistics, and Computer Science Department, Marquette University, Milwaukee, Wisconsin, United States of America; 3 Department of Epidemiology and Public Health, Swiss Tropical and Public Health Institute, Basel, Switzerland; 4 University of Basel, Basel, Switzerland; 5 Malaria Elimination Initiative, Global Health Group, University of California San Francisco, San Francisco, California, United States of America; 6 Divisions of Biostatistics and Epidemiology, University of California, Berkeley, California, United States of America; 7 School of Medicine, Tecnologico de Monterrey, Atizapan de Zaragoza, Estado de Mexico, Mexico; 8 Department of Global Health, University of Washington, Seattle, United States of America; Universidade Federal do Rio de Janeiro, BRAZIL

## Abstract

**Background:**

Despite great achievements by insecticide-treated nets (ITNs) and indoor residual spraying (IRS) in reducing malaria transmission, it is unlikely these tools will be sufficient to eliminate malaria transmission on their own in many settings today. Fortunately, field experiments indicate that there are many promising vector control interventions that can be used to complement ITNs and/or IRS by targeting a wide range of biological and environmental mosquito resources. The majority of these experiments were performed to test a single vector control intervention in isolation; however, there is growing evidence and consensus that effective vector control with the goal of malaria elimination will require a combination of interventions.

**Method and findings:**

We have developed a model of mosquito population dynamic to describe the mosquito life and feeding cycles and to optimize the impact of vector control intervention combinations at suppressing mosquito populations. The model simulations were performed for the main three malaria vectors in sub-Saharan Africa, *Anopheles gambiae s*.*s*, *An*. *arabiensis* and *An*. *funestus*. We considered areas having low, moderate and high malaria transmission, corresponding to entomological inoculation rates of 10, 50 and 100 infective bites per person per year, respectively. In all settings, we considered baseline ITN coverage of 50% or 80% in addition to a range of other vector control tools to interrupt malaria transmission. The model was used to sweep through parameters space to select the best optimal intervention packages. Sample model simulations indicate that, starting with ITNs at a coverage of 50% (*An*. *gambiae* s.s. and *An*. *funestus*) or 80% (*An*. *arabiensis*) and adding interventions that do not require human participation (e.g. larviciding at 80% coverage, endectocide treated cattle at 50% coverage and attractive toxic sugar baits at 50% coverage) may be sufficient to suppress all the three species to an extent required to achieve local malaria elimination.

**Conclusion:**

The Vector Control Optimization Model (VCOM) is a computational tool to predict the impact of combined vector control interventions at the mosquito population level in a range of eco-epidemiological settings. The model predicts specific combinations of vector control tools to achieve local malaria elimination in a range of eco-epidemiological settings and can assist researchers and program decision-makers on the design of experimental or operational research to test vector control interventions. A corresponding graphical user interface is available for national malaria control programs and other end users.

## Introduction

Despite great achievements by insecticide-treated nets (ITNs) [1] and indoor residual spraying (IRS) [2] in reducing malaria transmission, modeling and empirical evidence demonstrate that these tools are insufficient to eliminate malaria transmission in many settings today [3–6], even when combined with treatment with antimalarial drugs such as artemisinin combination therapies [7]. Protective coverage from indoor-based interventions is attenuated where mosquitoes can access blood resources from non-human hosts [8] or from humans when they are outdoors [9].

Fortunately, field experiments indicate that there are many promising vector control interventions that are underutilized or emerging and can be used to complement ITNs and/or IRS by targeting a wide range of biological and environmental mosquito resources [10]. The majority of these experiments were performed to test a single vector control intervention in isolation (or with ITNs and/or IRS); however, there is growing evidence and consensus that effective vector control will require a combination of interventions tailored to specific ecological and epidemiological settings [7, 10, 11].

We present a mathematical model that incorporates all stages of the mosquito life cycle from egg to larva, pupa and adult and, crucially, the female gonotrophic cycle whereby females blood feed and lay eggs. The goal is to provide an optimal package of vector control interventions required to reduce the entomological inoculation rate (EIR) to levels required for local elimination [12] for the three main African malaria vectors (i.e. *Anopheles gambiae s*.*s*. [13], *An*. *arabiensis* [14] and *An*. *funestus* [15]) in areas with low (EIR < 10 infective bites per person per year (pppy)), moderate (EIR = 10 − 100 bites pppy) or high transmission (EIR > 100 bites pppy).

The model is used to evaluate the impact of combining existing and novel interventions in synergistic ways in areas where ITNs and/or IRS are widely used but where malaria transmission persists. We consider the following vector control interventions in addition to ITNs and IRS: larviciding [16], attractive toxic sugar baits (ATSBs) [17], insecticide spraying of male mating swarms [18], mosquito-proofed housing [19], spatial mosquito repellents and other personal protection measures (e.g., topical repellents, insecticide-treated clothing) [20, 21], systemic and topical insecticide-treated cattle [22], odor-baited traps [23, 24] and space spraying [25]. Williams *et al* (*Unpublished*) provides a comprehensive review on the availability and quality of evidence of 22 vector control tools, including those included in this analysis.

The model presented here is used to quantify the impact of optimal packages with realistic intervention coverage on decreasing the mosquito feeding rate, reducing mosquito density, reducing the proportion of blood-meals taken from humans, extending the length of the gonotrophic cycle, reducing vectorial capacity, and reducing the basic reproduction number of the malaria parasite, *R*_0_. The main model output used to assess the impact of intervention is the entomological inoculation rate, *EIR*, which is the number of infectious bites per person per unit time, usually measured or expressed per year. Vector control interventions need to reduce EIRs to levels below 1 for a sustained period of time in order to interrupt malaria transmission [12]. We present the most comprehensive vector control optimization model (VCOM) to date that can be used by researchers and national malaria control programs (NMCPs) to develop potential vector control intervention combinations that can be tested to achieve malaria elimination in a range of eco-epidemiological settings. The model framework will be a useful tool in the selection of interventions for vector control trials and operational research and in the design of trials themselves especially for novel techniques.

## Methods

**Fig 1:**
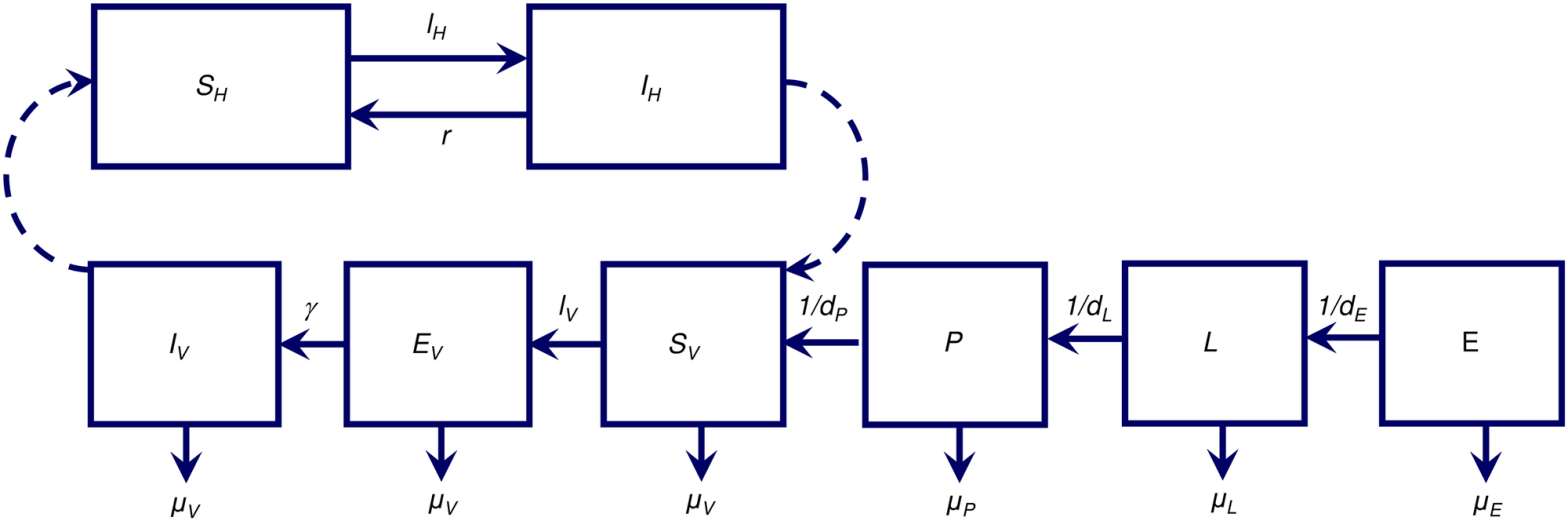
Flow diagram for the mosquito ecological model, mosquito SEI model, and human SI model. *E*, Early Instar; *L*, Late Instar; *P*, Pupae; *S*_*v*_, Susceptible Vectors; *E*_*v*_, Exposed Vectors, *I*_*v*_, Infected Vectors; *S*_*H*_, Susceptible Humans; and *I*_*H*_, Infected Humans.

We developed a modeling framework known as Vector Control Optimization Model (VCOM) to describe the population dynamics of mosquito vectors and to optimize the impact of combinations of interventions for the control and elimination of malaria transmission. The model is developed based on a compartmental model of mosquito life stages (i.e. progress from egg to larva to pupa to adult) and malaria infection amongst adult female mosquitoes using a Susceptible—Exposed—Infected/Infectious (SEI) framework. The human population is modeled using a simple Susceptible—Infected/Infectious (SI) model. The mosquito ecology and ITN/IRS models correspond to previous models [26–28] but with extensions to include a greater number of vector control tools. Full details of the model equations are presented in S1 Appendix and a schematic diagram representing the model is presented in Fig 1.

### Mosquito ecology and transmission models

Briefly, for the mosquito ecological model: (1) each female mosquito lays on average *β* eggs per day that hatch into early-instar larvae, *E*; (2) early-instar larvae are subject to density-dependent daily mortality at a rate *μ*_*E*_ and, if they survive, progress to late-instar larvae, *L*, after *d*_*E*_ days; (3) late-instar larvae undergo density-dependent daily mortality at a rate *μ*_*L*_ and, if they survive, develop into pupae (*P*) after *d*_*L*_ days; and (4) pupae undergo density-independent mortality at a rate *μ*_*P*_ or develop into adults, half of which are assumed to be females at emergence, *V*, after *d*_*P*_ days.

S1 Appendix provides detailed equations to elaborate the mosquito life and feeding cycle model, which describes the factors contributing to the rate at which female mosquitoes feed on human and non-human hosts and lay eggs in the absence of interventions. Factors described in the model include the time to complete one feeding cycle, the female mosquito death rate and the total adult female mosquito population size, which is a function of density-dependent competition at the larval stage and the carrying capacity of the environment for larvae. Malaria infection is incorporated into the mosquito population dynamics by subdividing the adult female mosquito population (*V*) into those susceptible (*S*_*V*_), latently infected (*E*_*V*_), and infectious (*I*_*V*_) for the malaria parasite.

Once a susceptible mosquito bites an infectious human, then the mosquito moves into the exposed/latently infected stage, *E*_*V*_, at a rate equivalent to the force of infection in vectors, *λ*_*V*_. After completing a latent infection period of fixed duration, *τ* = 1/*γ*, a fraction of mosquitoes, $e^{-\mu_{V}\tau }$, survive this latent period to become infectious, *I*_*V*_. Here, *μ*_*v*_ represents the daily natural mortality rate that adult female mosquitoes undergo, which is assumed to be the same independent of infection status.

### Malaria infection in the human population model

As we are focusing on the mosquito population dynamics, we describe malaria infection in the human population using a simple susceptible-infectious (SI) model—a susceptible human (*S*_*H*_) becomes infected (*I*_*H*_) at a rate equivalent to force of infection in humans, *λ*_*H*_. Infected humans are assumed to recover from infection at the human recovery rate, *r*.

### The impact of interventions—Targeting mosquitoes when using their resources

In order to achieve malaria elimination, strategies are needed that target mosquitoes when they evade ITNs/IRS and feed on humans indoors, feed on animals or humans outdoors, or while using one of the other biological or environmental resources they use, such as sugar, mating sites, resting sites and oviposition sites. With this in mind, we extend the above model to incorporate interventions that target mosquitoes both indoors and outdoors and at all stages of the mosquito life and feeding cycles.

Upon emergence from eggs, female mosquitoes mate and sugar feed—life processes which may be targeted by spraying of male swarms and ATSBs, respectively. The mosquito gonotrophic cycle then follows, in which female mosquitoes start host-seeking—the process that can be targeted by space spraying with insecticides and odor-baited traps—and then alternate between blood-feeding (in order to support egg production) and egg-laying. There are multiple options that female mosquitoes may pursue to obtain a blood meal: a) biting livestock, which can be mitigated by treating livestock with endectocides (antiparasitic drugs that act as a systemic insecticide) or spraying livestock with insecticides; b) biting humans outdoors, against which humans can protect themselves with spatial repellents and other personal protection measures; and c) biting humans indoors, for which ITNs, IRS, spatial repellents and mosquito-proofed housing are protective. After taking a blood-meal, female mosquitoes then produce eggs and oviposit (lay eggs) in water. Effective interventions targeting this stage of the life cycle include larviciding (conventional or aerial), biological control and larval source reduction, since they all kill immature mosquito forms in aquatic habitats. Mosquitoes can also be targeted while seeking humans, resting sites and oviposition sites using ovitraps, ATSBs and space spraying with insecticides.

**Fig 2:**
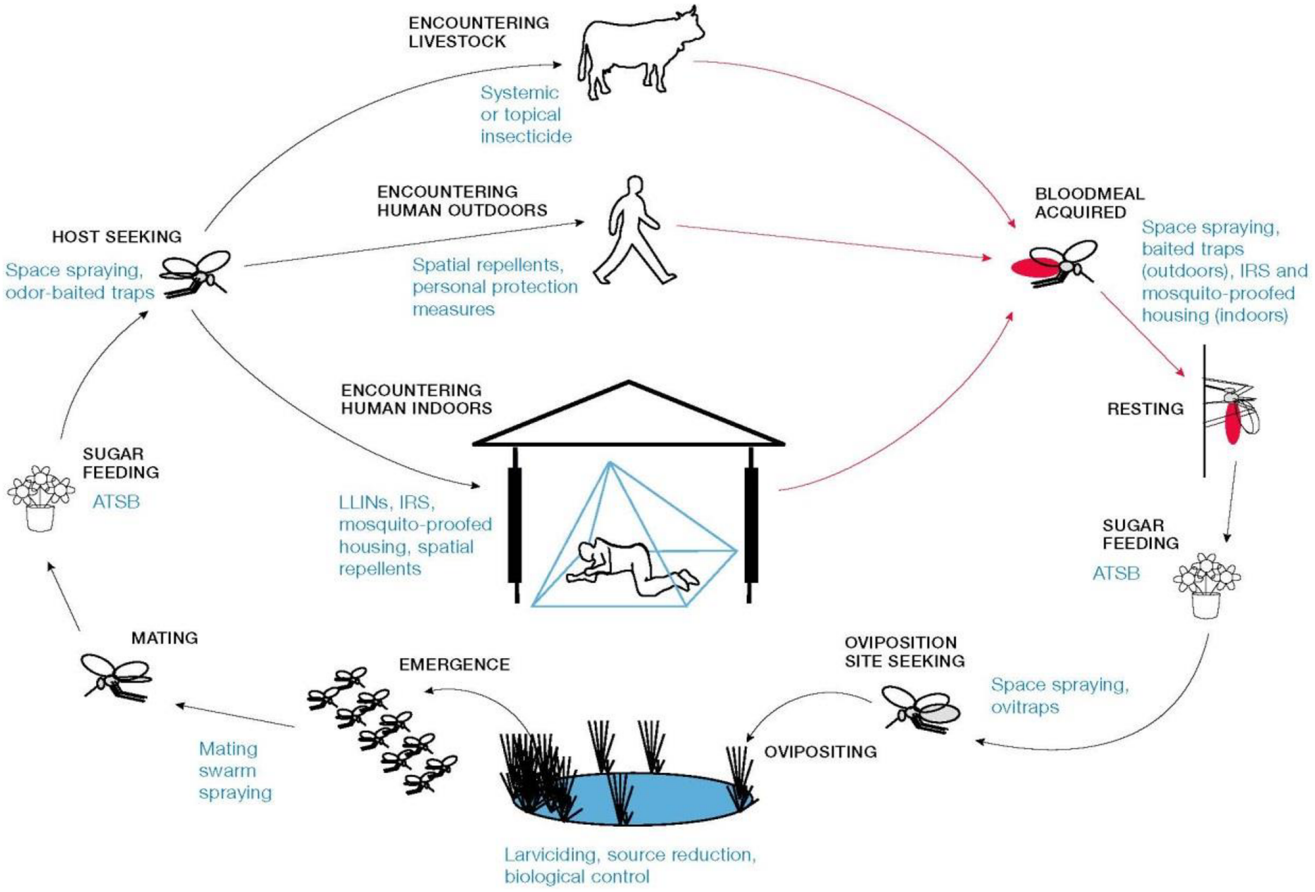
Targeting the mosquito on multiple fronts. The schematic highlights opportunities for existing and novel vector control tools that can be used to target mosquitoes both indoors and outdoors and at all stages of the mosquito life and feeding cycles. Synergy and layering between interventions follows from this schematic diagram (Fig 2) and how all the interventions are encoded from it.

These interventions are modeled by the effects they have on diverting mosquitoes, which increases the amount of time it takes for the gonotrophic cycle to be completed, and hence decreases the mosquito feeding/biting rate. Other effects which are modeled are: a) the effect of interventions on the mosquito daily mortality rate, which impacts the total mosquito population size; and b) the effect of these interventions on reducing the human biting rate, which is relevant for malaria transmission from both mosquito-to-human and human-to-mosquito. We therefore describe different vector control interventions that can be used to target mosquitoes while utilizing a given resource. The mathematics of the model detailed follows from the schematic shown in Fig 2, highlighting several opportunities for vector control.

In the next subsections, we describe briefly how each intervention is implemented. Full mathematical details are provided in S1 Appendix, and a complete table of parameter definitions and range of values used for model simulations are provided in Tables, A, B, and C in S2 Appendix.

### Targeting mosquitoes in aquatic habitats

We consider three interventions that can be used to target the immature mosquito forms and eventually reduce the mosquito emergence rate—an effective vector control approach targeting all mosquito species regardless of their behavior. These are source reduction, larviciding, and biological control.

#### The impact of source reduction in reducing environmental carrying capacity

Environmental management through source reduction targeting mosquito aquatic stages complements existing adult control measures well [29]. We incorporate the impact of source reduction in the model by considering its impact on environmental carrying capacity, *K*. This impact is modeled by considering the effectiveness of the source reduction approach used (e.g. drainage of canals, eliminating ditches) and the proportion of aquatic habitats where source reduction is implemented in reducing the value of *K*. As noted previously [30], source reduction is most effective in urban or developed areas where it is easier to identify, map and treat mosquito breeding sites.

#### The impact of larviciding and biological control in suppressing the immature mosquito population

The impact of larviciding and biological control is modeled by the impact it has on early instars, late instars, and pupae which reside in aquatic habitats where these interventions are applied alone and/or in combination. The early and late instar and pupal stages–*E*, *L* and *P*–are divided into *E*_*l*_, *E*_*bc*_, *E*_*l*,*bc*_, *L*_*l*_, *L*_*bc*_, *L*_*l*,*bc*_, *P*_*l*_, *P*_*bc*_ and *P*_*l*,*bc*_ to represent immature stages in breeding sites where larviciding and biological control [29] are applied alone and in combination for the three mosquito stages respectively (the subscript *l* represents larviciding alone, *bc* represents biological control alone, *l*, *bc* represents both, and *l*, *bc*, 0 represent neither). The effectiveness of each intervention at a given coverage is captured by its ability to increase the death rate of immature forms by multiplying these by a factor allowing for increased death rate due to biological control, *ζ*_*bc*_, or larviciding, *ζ*_*l*_. It is assumed that the two interventions act independently to each other so that the impact of combined interventions is obtained by multiplying the two factors allowing for increased death rate together, i.e. *ζ*_*l*_ *ζ*_*bc*_.

### Targeting mosquitoes while mating

After emergence, female mosquitoes mate in flight with male mosquitoes, which normally gather in swarms at specific mating sites over landmarks [18]. We can target male mosquito swarms by increasing the male mosquito death rate using targeted space spray with insecticides. As the number of adult male mosquitoes decreases, it becomes more difficult for adult female mosquitoes to find a mate, and hence the egg-laying rate of adult females decreases. In order to incorporate this intervention, we have used a simple sigmoidal function to describe the relationship between the egg-laying rate of female mosquitoes and the ratio of male and female adult mosquitoes.

### Targeting mosquitoes while searching for blood

Mosquitoes can be targeted while host seeking by using ATSBs, space spraying and/or odor baited traps. These interventions can all increase the mosquito death rate, hence decreasing the probability of a mosquito surviving the host-seeking process and resulting in a decline in the mosquito population size.

#### Estimating the impact of attractive toxic sugar baits and space spraying on host-seeking mosquitoes

Mosquitoes feed frequently on plants and other sugar sources, providing an opportunity to attract and kill them using ATSB-sprayed plants or other baits while host-seeking outdoors. Sugar is a major source of energy for female mosquitoes and the sole energy source for males [31, 32]. The impact of ATSBs on female mosquitoes is modeled by their ability to increase mosquito death rate by a known factor *ζ*_*ATSB*_ when mosquitoes feed on ATSB-sprayed plants. On the other hand, space spraying can be used to target outdoor host-seeking mosquitoes by increasing mosquito natural death rate by a factor, *ζ*_*SS*_. The impact of these interventions is computed when each intervention is used alone or in combination assuming independent coverage of each intervention.

#### Estimating population level effects of odor-baited traps

The efficacy of odor-baited traps is derived primarily from two complementary characteristics: a) their high attractiveness to mosquitoes compared to attractiveness of humans; and b) their ability to trap and kill mosquitoes which attack them hoping to obtain blood, thus removing these mosquitoes from the host-seeking mosquito population [23, 24]. Thus, odor-baited traps also affect the probability of a mosquito surviving the host-seeking process. The impact of odor-baited traps is modelled by the impact it has in preventing a proportion of mosquito bites that would otherwise occur on humans or non-human hosts. This is modeled by considering: a) the availability of one trap in relation to one human, *α*_*t*_, and b) the ratio of traps to humans, *r*_*t*_.

### Targeting mosquitoes while attempting to feed on humans indoors

We describe the impact of indoor interventions, specifically, insecticide treated nets (ITNs), indoor residual spraying (IRS) and housing modification (HM), following the approach of Le Menach *et al*. [27]. It is assumed that a mosquito can be killed when encountering ITNs or IRS and repelled when encountering any of these three interventions. Repellency results in a longer gonotrophic cycle and hence a slower egg-laying rate and consequently a smaller adult mosquito population size. All modelled house modifications are assumed to repel mosquitoes; but the ability of housing modifications to kill mosquitoes depends on the type of housing modification being implemented—for example houses with treated netting eave baffles are assumed to both kill and repel mosquitoes.

For all three interventions, a mosquito can successfully feed with a given probability, *s*. The impact of a house with a single intervention or combination of any of two or three is modeled by the potential of the interventions to prevent mosquitoes from feeding by repelling or killing them, as illustrated in Figure C in S1 Appendix. The impact of each intervention is computed, on its own or in combination, assuming independent coverage of each. The impact is also assumed to remain the same throughout the duration of the simulation. The proportion of bites taken by a given vector species indoors is considered, and further divided into the proportion of bites taken on people in bed and otherwise indoors.

### Targeting mosquitoes while attempting to feed on humans when outdoors

Mosquitoes attempting to bite humans outdoors can potentially be blocked from biting these humans by spatial repellents and other personal protection measures (e.g. topical repellents and insecticide-treated clothing). The impact of these interventions is computed when each intervention is used alone or in combination, assuming independent coverage of each and its potential to repel and prevent mosquitoes from feeding on unprotected humans outdoors.

### Targeting mosquitoes while attempting to feed on non-human hosts

Several experiments [22, 33, 34] have indicated that endectocide-treated cattle can reduce malaria transmission in humans. We explore the impact of systemic and/or topical insecticide applied to cattle by increasing the mosquito mortality rate associated with insecticide contact during blood-feeding from cattle. The impact of insecticide-treated cattle is modeled for interventions applied to cattle in isolation or combination.

### Targeting mosquitoes while seeking resting sites, resting, and searching for oviposition sites

Mosquitoes can be targeted while searching for resting or oviposition sites, and resting after acquiring blood-meals, by ATSBs, space spraying (SS) and/or other traps (e.g. ovitraps). Each of these interventions increases the mosquito death rate, in this case, during the second phase of the gonotrophic cycle (after blood-feeding, before egg-laying). The impact of ATSBs on female mosquitoes is modeled by their ability to increase mosquito death rate by a known factor, *ζ*_*ATSB*_, when mosquitoes feed on ATSB-sprayed plants. Space spraying can be used to target outdoor resting mosquitoes by increasing the natural death rate of mosquitoes by a factor of *ζ*_*SS*_. Finally, ovitraps target ovipositing mosquitoes, hence they have two impacts on the mosquito population: a) increasing the adult female death by a factor *ζ*_*OT*_; and b) reducing the number of viable eggs that a female mosquito lays per oviposition cycle.

### Model output

We present all the primary and secondary model outputs that can be generated by the model to assess the impact of combined interventions in different transmission settings. Mathematical details of the model outputs are also presented in S1 Appendix.

#### The impact of combined interventions on mosquito population parameters

We assessed the impact of the above described interventions in terms of: a) extending the length of gonotrophic cycle due to mosquitoes being repelled while attempting to feed on humans indoors and outdoors and upon cattle; b) reducing the probability of mosquitoes surviving for one day and hence increasing their death rate; c) reducing the number of viable eggs laid per day per female mosquito by considering the number of viable eggs that a female mosquito lays per oviposition cycle, the length of gonotrophic cycle and the mosquito daily death rate; d) reducing the proportion of blood-meals obtained from humans by taking the ratio of successful blood-meals taken upon humans over successful blood-meals on all hosts; and e) reducing the human biting rate per mosquito by dividing the proportion of blood-meals obtained from humans over the length of gonotrophic cycle.

#### Estimating the entomologic inoculation rate (EIR) in the presence of interventions

The main model output is malaria transmission intensity, often expressed in terms of the entomologic inoculation rate (EIR) which is a direct, practical indicator of human exposure to bites of mosquitoes infected with transmissible sporozoite-stage malaria parasites. In other words, EIR is the number of infectious bites per person per unit time, usually measured or expressed per year. It is important for vector control interventions to sustainably reduce EIRs to levels below 1 in order to interrupt malaria transmission [12, 35]. Therefore, the impact of optimal intervention packages is modeled on their potential to reduce annual EIR below one.

#### Estimating vectorial capacity in the presence of interventions

Vectorial capacity is another important parameter which can be used to estimate the risk of malaria introduction. Dye [36] defines vectorial capacity as the daily rate at which future inoculations arise from currently infective humans, assuming that all female mosquitoes biting that infected human become infected. The model can also be used to assess the impact of combined intervention in reducing the vectorial capacity.

#### Estimating the basic reproductive number in the presence of interventions

The last model output is the basic reproductive number of the malaria parasite, *R*_0_, which represents the number of new human infections that one human case introduces to a susceptible population on average over the course of its infectious period, 1/*r*. *R*_0_ is an important indicator because it specifies the threshold for continued disease transmission (i.e. if *R*_0_ < 1, the infection will disappear in the long run, and if *R*_0_ > 1, the infection will be able to spread in the population). The higher the value of *R*_0_, the harder it is to control the infection in the population [37]. Our model can be used to simulate combinations of interventions and specific coverage values and infer which combination interventions lead to values of *R*_0_ less than one.

### Model simulations

The model simulations are implemented based on populations of *Anopheles gambiae s*.*s*., *An*. *arabiensis*, and *An*. *funestus* mosquitoes. The model results presented here are obtained by assessing the impact of combined interventions at different coverage levels in reducing malaria transmission in areas with low (EIR < = 10), moderate (10 < EIR < = 100) and high (EIR > 100) transmission to an extent required for local malaria elimination (i.e. to low values of EIR required to interrupt malaria transmission such that *R*_0_ < 1). The model was used to sweep through parameter space to predict combined interventions with specific coverage levels that can be used to reduce annual EIR below 1 in a range of transmission settings noted above. The model simulations for different intervention scenarios are run until equilibrium is reached and the output values are reported at equilibrium.

### Sensitivity analysis

We performed sensitivity analysis to determine which parameters have great influence on the model output i.e., entomological inoculation rate, based on 50% coverage of ITNs, attractive toxic sugar baits (ATSB), systemic (ECS) and topical (ECT) endectocide-treated cattle, mosquito-proofed housing (HOU), larviciding (LAR) and personal protection measure (PPM). The same selected tools are used in providing sample model results as presented in the results section. We have used the Latin Hypercube Sampling/Partial Rank Correlation Coefficient (LHS/PRCC) sensitivity analysis approach to explore the entire parameter space of the model based on the selected interventions with their corresponding parameter values. A wider range is assigned to parameter values as presented in Table C in S2 Appendix where data is unavailable.

**Fig 3:**
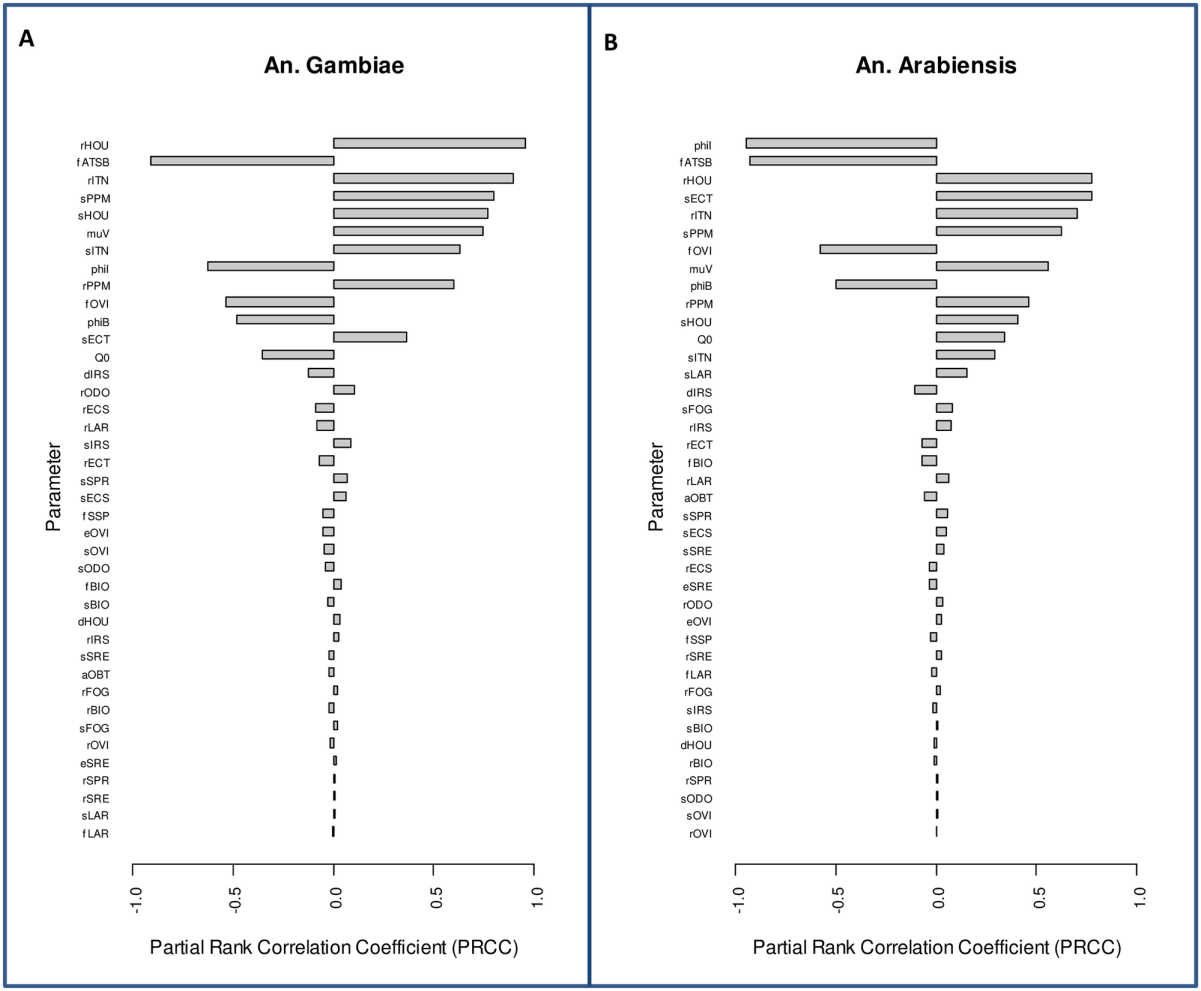
Sensitivity analysis for vector control optimization model. Sensitivity analysis is performed using Latin Hypercube Sampling/Partial Rank Correlation Coefficient (LHS/PRCC) sensitivity analysis approach based on the impact of selected vector control tools at 50% coverage in reducing entomological inoculation rate for *An*. *gambiae s*.*s*. (**A**) and *An*. *arabiensis* (**B**). The names for parameters are given in the second column titled “Key” of Table C in S2 Appendix.

LHS/PRCC sensitivity analysis can identify and rank key parameters according to their importance in contributing toward model prediction imprecision. The closer the absolute value of the PRCC is to one for a particular parameter, the more influence that parameter has on the model output. More details on LHS/PRCC sensitivity analysis approach can be found here [38, 39]. To compute the PRCC values for our model, we use the implementation of LHS and PRCC functions in R [40] version 3.2.4 –the parameter values used were drawn from a uniform distribution. The functions were run on 1000 samples for a total of 40 parameters based on the range of values given in Table B in S2 Appendix as minimum and maximum values required by the uniform distribution. As indicated in Figure C in S1 Appendix, probability of mosquito being repelled upon encountering a mosquito proofed housing (*rHOU*) (A) and insecticide treated nets (*rITN*) (A and B), a probability of feeding and surviving upon encountering a mosquito proofed housing (*sHOU*) (A) and a personal protection measure (*sPPM*) (A and B), mosquito death rate while searching for blood (*muV*) (A), a proportion of mosquito bites on a person while they are indoor (*phiI*) (A and B), a factor allowing for increased mosquito death rate due to ATSB (*fATSB*) (A and B), and a probability of feeding and surviving upon encountering a topical endectocide-treated cattle (*sECT*)—all have the most influence on the model output for *An*. *gambiae s*.*s*. and *An*. *arabiensis* as indicated in Fig 3 panels A and B. Sensitivity analysis results for *An*. *Funestus* are represented in Figure D in S1 Appendix.

### GUI/Software

VCOM’s graphical user interface (GUI) is freely available online (http://skiware.github.io/VCOM/) for users to explore the impact of combination interventions of their own interest. Users are able to run simulations based on a given mosquito species, transmission setting and choice of intervention(s). Advanced options are available to alter the parameter values of the mosquito ecology and intervention models as desired. A short summary of the GUI is provided in S3 Appendix. The VCOM code is developed in R version 3.2.4 and the link to the GitHub repository is provided in the GUI’s introduction page.

## Results

The VCOM framework presented here is capable of exploring a range of eco-epidemiological settings with differing transmission intensities, vector species and relative densities of non-human hosts. In this section, we present limitations of the current indoor tools (i.e. ITNs and IRS) and sample results on the optimal intervention packages incorporating ITNs required to interrupt malaria transmission (i.e. to reduce annual EIRs to levels below 1) for a population of *Anopheles gambiae s*.*s*., *An*. *arabiensis* and *An*. *funestus* mosquitoes in areas with low (EIR = 10), moderate (EIR = 50) and high malaria transmission (EIR = 100).

### ITNs or IRS alone are unlikely to eliminate malaria

Using mathematical models to evaluate the actual impact of ITNs based only on reported coverage is challenging due several factors such as adherence to ITNs use, decay in insecticide applied to ITNs effectiveness over time, insecticide-resistance among the local vector population, and uncertainty regarding the actual coverage level of these interventions following roll-out. Furthermore, models describing the impact of these interventions often overestimate their impact as they are frequently parameterized based on successful field trials, which may not be representative of all roll-out settings.

**Fig 4:**
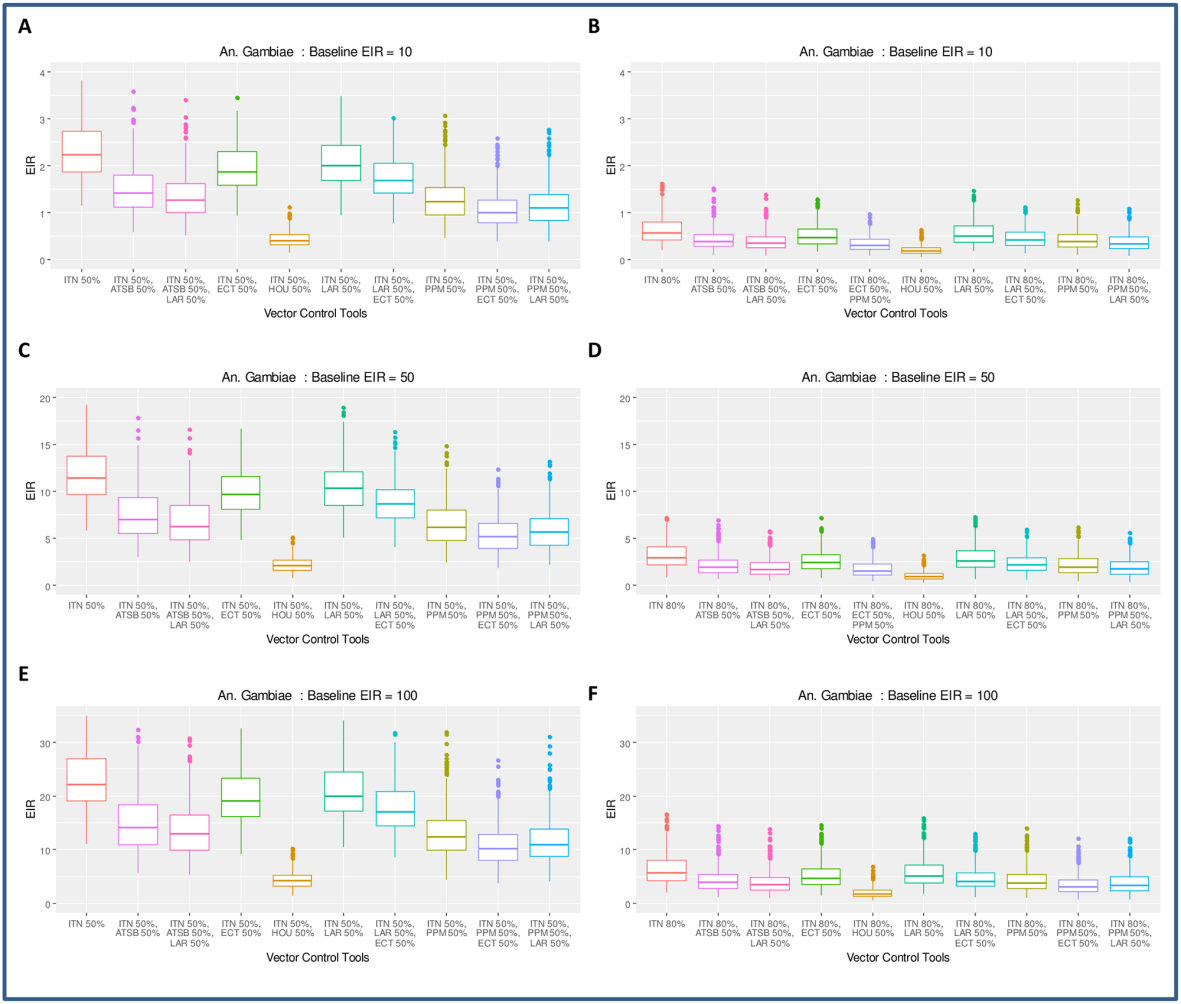
Evaluating the impact of combining ITNs at 50% and 80% coverage with additional tools against *An*. *gambiae s*.*s*. The tools selected in this example are attractive toxic sugar baits (ATSB), topical (ECT) endectocide-treated cattle, mosquito-proofed housing (HOU), larviciding (LAR) and personal protection measure (e.g. insecticide-treated clothing) (PPM). Adding one or two tools to ITNs at 50% coverage might be sufficient to interrupt transmission in low transmission settings (**A**); but in most cases not sufficient in moderate (**C**) and high transmission (**E**) settings. Scaling up ITNs to 80% coverage and adding another tool with 50% coverage might be sufficient to interrupt transmission in low (**B**) transmission but not necessarily in moderate (**D**) and high (**F**) transmission settings unless the tool added is mosquito proofed housing.

**Fig 5:**
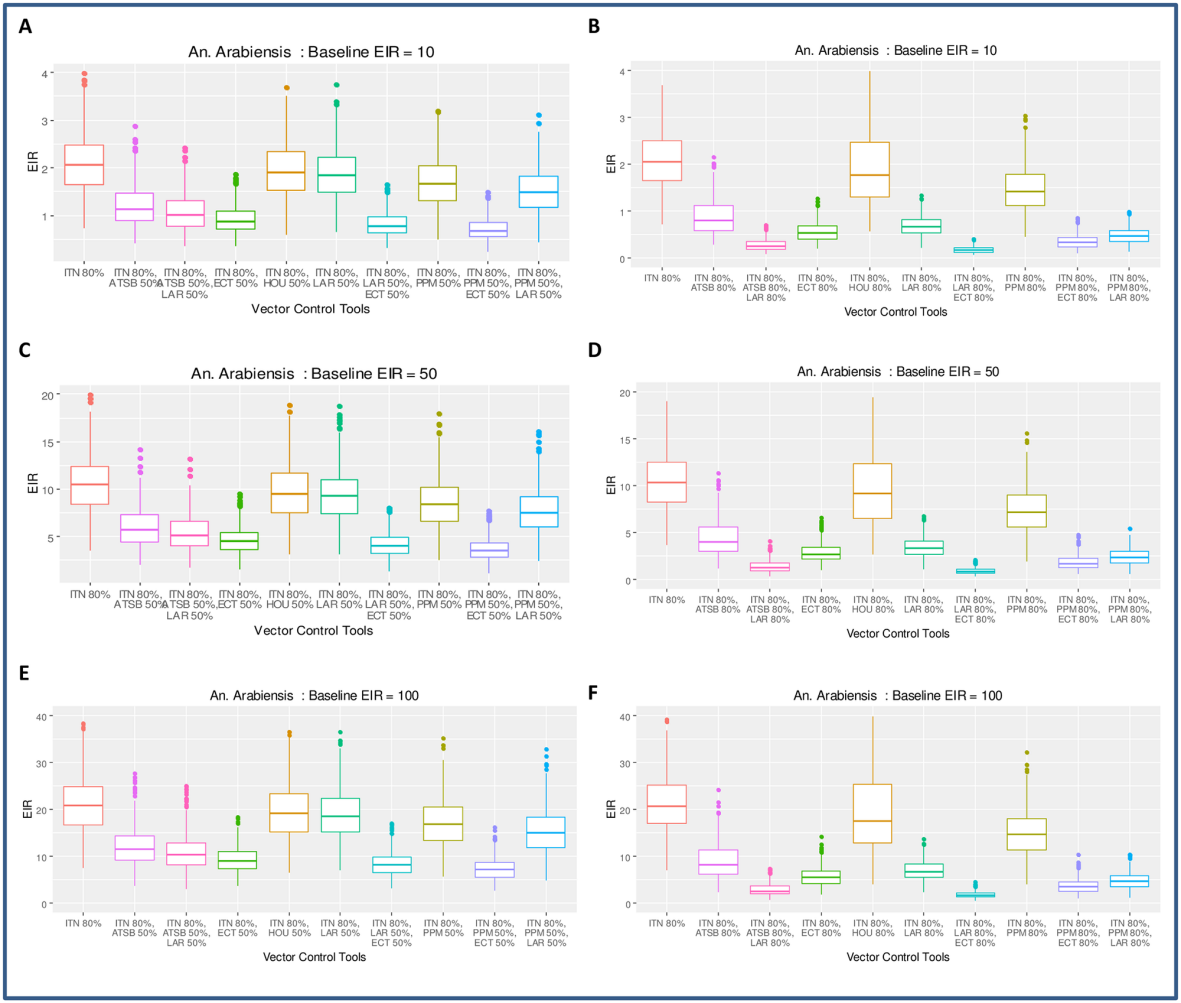
Evaluating the impact of combining ITNs at 80% coverage with additional tools against *An*. *arabiensis*. Similar tools presented in Fig 4 are presented here showing EIR values at equilibrium but with ITN coverage set to 80% and coverage with additional vector control tools set to 50% in panels A, C and E and 80% panels B, D and F. Adding a tool or two is insufficient to interrupt transmission in most cases for *An*. *arabiensis* in moderate and high transmission settings, except when larviciding or endocticide-treated cattle is added (either alone or in combination).

However, even given these overestimates of impact and setting the ITN coverage levels to 80%, the model indicates that it might be possible to interrupt malaria transmission with ITNs alone in low transmission settings (baseline EIR of 10) for *An*. *gambiae s*.*s*. (Fig 4B) and *An funestus* (Figure E panel B in S1 Appendix); but ITNs will be insufficient to interrupt transmission in moderate transmission settings (baseline EIR of 50) (Figs 3D and 4D and Figure E panel D in S1 Appendix) and in high transmission settings (baseline EIR of 100) (Figs 4F and 5F and Figure E panel F in S1 Appendix) for any of the three malaria vectors. The model indicates that it might not be possible to interrupt transmission with ITNs alone based on 50% for any of the three vectors in any of the transmission settings—results depicting this are presented in Figs 4 and 5 and Figure E in S1 Appendix in panels A, C, and E for ITN coverage at 50%.

### Evaluating the impact of combining ITNs at 50% and 80% coverage with additional tools to control *An*. *gambiae s*.*s*.

The model is used to investigate additional interventions to ITNs at 50% and 80% coverage that would be successful in interrupting malaria transmission for vector populations predominated by *An*. *gambiae s*.*s*. in all the three transmission settings (Fig 4). Fig 4 shows equilibrium values of EIR at 50% or 80% coverage of ITNs and after adding selected tools to areas with low (**A** and **B**), moderate (**C** and **D**), and high (**E** and **F**) malaria transmissions. The box plot shows the median, the interquartile ranges and the 95% confidence ranges of the EIR for different parameter values shown on Fig 3 corresponding to either specific mosquito parameters and/or vector control tools used to interrupt transmission.

As shown in Fig 4, adding one and even a second intervention at 50% coverage might not be sufficient to interrupt high (panel **E**) or moderate transmission (panel **C**). On the other hand, adding mosquito proofed housing (HOU) at 50% might be sufficient to interrupt low (panel **A**) transmission against *An*. *gambiae s*.*s*. By scaling up ITNs to 80% coverage and then adding any of the selected intervention at 50% should be sufficient to interrupt transmission against *An*. *gambiae s*.*s*. in areas with low (panel **B**) malaria transmission. Only adding mosquito proofed housing seems to interrupt transmission in areas with high (panel **F**) and moderate (panel **D**) malaria transmission. Equivalent model simulations performed for *An*. *funestus* predict slightly different results presented in Figure E in S1 Appendix.

### Evaluating the impact of combining ITNs at 80% coverage with additional tools to control *An*. *arabiensis*

Simulations for *An*. *arabiensis* conducted with ITNs coverage at 50% indicate ITNs are much less impactful against *An*. *arabiensis* due to their preference to feed on both humans and animals, and their proclivity to feed outdoors.

We therefore opted for simulations with 80% ITNs coverage for *An*. *arabiensis* and additional interventions at either 50% coverage (Fig 5, panels **A**, **C** and **E**) or 80% coverage (Fig 5 panels **B**, **D** and **F**). Model results presented in Fig 5 suggest that, in most cases, adding one or two tools at 50% coverage (in addition to ITNs at 80% coverage) might not be sufficient to interrupt malaria transmission for *An*. *arabiensis* populations (Fig 5 panels **A**, **C**, and **E**). Model predictions suggest that the most effective way to control *An*. *arabiensis* is to treat cattle with insecticide and also target aquatic habitats with larviciding Fig 5 (panels **B**, **D**, and **F**) while maintaining ITN coverage at 80%.

### Evaluating the impact of combining ITNs at 50% coverage with interventions not requiring human participation

**Fig 6:**
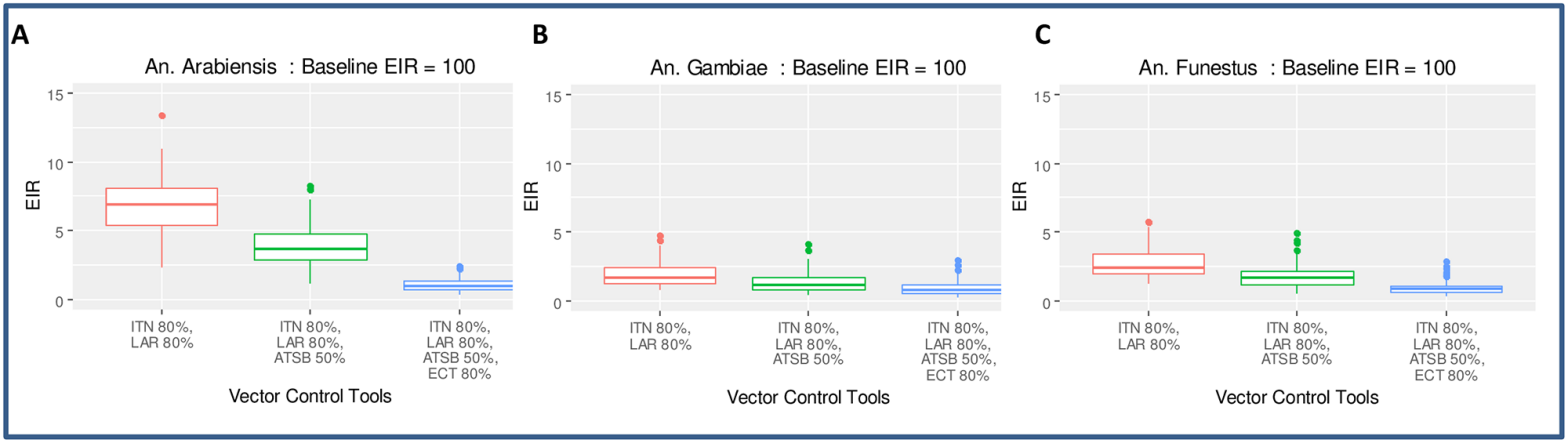
Evaluating the impact of combining ITNs at 80% coverage with other interventions. This figure shows equilibrium values of EIR simulated based on selected tools added to high transmission areas against *An*. *arabiensis* (**A**), *An*. *gambiae* (**B**), and *An*. *funestus* (**C**). The box plot shows the median, the interquartile ranges and the 95% confidence ranges of the EIR for different parameter values shown on Fig 3 corresponding to either specific mosquito parameters and/or vector control tools used to interrupt transmission. Adding larviciding at 80% to ITNs at 50% might not be sufficient to interrupt transmission (**A**, **B**, and **C**); but adding another intervention (e.g. ATSB at 50%) reduces transmission dramatically and adding the fourth intervention (e.g. endectocide-treated cattle at 80%) is sufficient to interrupt transmission with baseline EIR of 100.

We evaluated combining ITNs at 80% coverage with interventions that do not depend on human participation. Considering that human participation can impede the impact of vector control tools (e.g. people do not sleep under ITNs or refused to have their homes sprayed with insecticide), we ran simulations including ITNs and additional selected interventions that target non-human hosts (endocticide-treated cattle), the larval habitat (i.e. larviciding) both at 80%, and sugar sources (i.e. ATSB) at 50%. As shown in Fig 6, adding larviciding at 80% to ITNs at 50% might not be sufficient to interrupt transmission in high (EIR = 100) transmission settings. This combination of interventions has the lowest impact on *An*. *arabiensis* (panel **A**) given their feeding preferences mentioned above. Adding ATSB as the third intervention dramatically reduces transmission, and adding topical endectocide-treated cattle (ECT) is predicted to interrupt transmission for all the three studied species. Thus, the selected tools which do not require human compliance are effective against all the three species. Since they are effective in high transmission settings (baseline EIR of 100), they should also be effective in low and moderate transmission settings for all the three studied species, even at the coverage levels reported here.

## Discussion

The model presented here can be used to examine optimal vector control intervention packages to support the design of operational and experimental research and implementation to reduce and eliminate malaria transmission.

The findings from our analyses indicate that ITNs alone will likely be insufficient to interrupt transmission, except with a predicated coverage of 80% in low transmission settings, which is the hypothetical maximum level of coverage compared to the reported 55% coverage in sub-Saharan Africa in 2015 [41]. More likely, interventions in addition to ITNs will be required, especially for *An*. *arabiensis*, due to the vector’s proclivity to feed on non-human hosts. Additional interventions including endectocide-treated cattle and larviciding were predicted to reduce *An*. *arabiensis* transmission to low levels. Importantly, we have presented optimal intervention combinations with interventions not requiring human participation, limiting the reliance on human adherence. As one would expect, the more interventions that were added to each simulation, the lower the predicted transmission. That said, larviciding and ATSBs and/or endectocide-treated livestock were predicted to have the most significant impact on transmission across vector species and transmission intensity. However, achieving high coverage (e.g., 80%) of larviciding using classic conventional method might be a very serious challenge and unlikely operation wise, hence, aerial larviciding might be an ideal alternative approach in reaching high coverage of aquatic habitats with larvicide.

The results from our model are similar to previous model simulations and field experiments [7, 42–44]. In all cases, high coverage with indoor interventions has been reported to be more effective against *An*. *gambiae s*.*s*. and *An*. *funestus* but not *An*. *arabiensis*. Also, the recommendation to use insecticide-treated cattle to control malaria transmission vectored by *An*. *arabeinsis* is similar to the previous studies [33].

Previous studies [3, 5, 7, 45–47] have recommended that novel tools be considered to complement ITNs, and have emphasized the need to quantify the overall impact of these novel tools to control malaria transmission. Our model can be used to quantify a comprehensive list of vector control tools in addition to ITNs. Simulation results presented here are examples of scenarios in which combinations of interventions may be effective in targeting indoor and outdoor biting and resting mosquitoes, mosquito feeding on non-human hosts, feeding on sugar sources, and at larval aquatic stages. It is clear from the results that targeting mosquitoes at aquatic stages with either larviciding, biological control or source reduction is a highly effective option.

As with any modeling analysis, there are limitations such as excluding several factors relevant to malaria transmission, including seasonality, heterogeneity and mosquito resistance against insecticides. As an example, implication of ignoring seasonality would be larviciding may achieve lower levels of coverage during the rainy season so would be less effective; or interventions with short half-lives such as IRS could be more effective. Seasonality is an important aspect that we will investigate further in future work as an expansion of VCOM framework. Also, insecticide resistance threatens the efficacy of insecticide-based interventions and is an issue that should be considered when evaluating the impact of these interventions. Despite these limitations, the VCOM framework can be a highly useful decision-making tool in the design of vector control strategies and will provide increasingly accurate insights and decision support as development continues. The modeling framework allows the synergies of a range of interventions to be captured and for the combined impact of various combinations of interventions to be predicted in a range of settings. Similarly, VCOM will be a useful tool in the selection of interventions for vector control trials and operational research and in the design of trials themselves. Although, operational predictions cannot be made using this framework due to the lack of model detail and data to inform such model detail, the relative scale of intervention impact predicted by the VCOM framework provides programmatically useful guidance on which interventions are most synergistic in a range of settings. Using this modeling framework, researchers and national malaria control programs (NMCPs) will be able to explore intervention combinations for malaria elimination in a range of eco-epidemiological settings in areas with low, moderate, and high malaria transmission.

In the absence of detailed data on correlations between parameters, for the sensitivity analysis, we make the standard assumption that parameters are independently distributed with uniform distributions. As new data become available from novel and existing interventions, the VCOM framework will be refined, parameterized and validated. We will be able to use the framework to provide insights on optimized interventions packages that can be used to target zoophagic vectors and/or multi-species populations at once. Also, the development of resistance by mosquito species against vector control tools will be incorporated. Through the addition of cost data, cost-effectiveness analysis will be performed in a range of settings of interest to malaria control programs.

The VCOM modeling framework is available as an easy-to-use online platform with expectations that this will enhance the accessibility and usefulness of the modeling framework. Nonetheless, VCOM is a simplification of complex dynamical systems for malaria transmission, control and elimination, so recommendations from VCOM should be considered more as providing insights rather than firm, quantitative predictions. Researchers should work closely with NMCPs and other stakeholders and end users to ensure these tools provide useful insights and can assist in informed decision making.

## Supporting information

S1 Fig Model schematic diagram. Flow diagram for the mosquito ecological model, mosquito SEI model, and human SI model.
(TIF)

S2 Fig Targeting mosquito on multiple fronts. The schematic highlights opportunities for vector control to target biological and environmental mosquito resources.
(TIF)

S3 Fig Illustrations of the impact of indoor interventions. The schematic illustrates the probability of mosquito being repelled or killed upon encountering indoor interventions.
(TIF)

S4 Fig Sensitivity analysis results for *An*. *funestus*. (TIF)

S5 Fig Evaluating the impact of combining ITNs at 50% and 80% coverage with additional tools against *An*. *funestus*. (TIF)

S6 Fig Illustrations of VCOM’s simple graphic user interface. (TIF)

S1 Table Parameter values for the vector and human components of the model. (DOCX)

S2 Table Basic parameter estimates for the vector ecology and control model. (DOCX)

S3 Table Parameter for the effectiveness of all interventions considered. (DOCX)

S1 Appendix Full details of the mathematical models. (DOCX)

S2 Appendix Brief description of the model parameter values. (DOCX)

S3 Appendix VCOM software—User graphical interface. (DOCX)
