# Supplementary figures and images for: Attacking the mosquito on multiple fronts: Insights from the Vector Control Optimization Model (VCOM) for malaria elimination
Source: PLoS One. 2017 Dec 1;12(12):e0187680. doi: 10.1371/journal.pone.0187680 (PMC5711017; doi:10.1371/journal.pone.0187680)

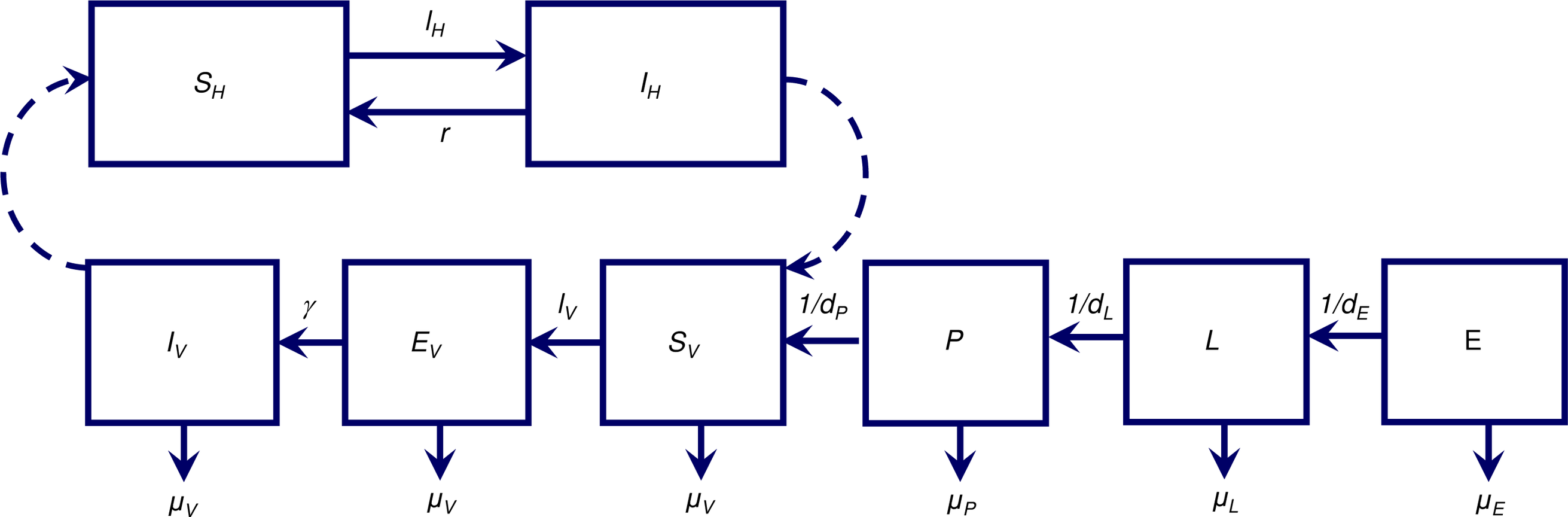

Supplement: S1 Fig — Flow diagram for the mosquito ecological model, mosquito SEI model, and human SI model. (TIF) [file pone.0187680.s001.tif]

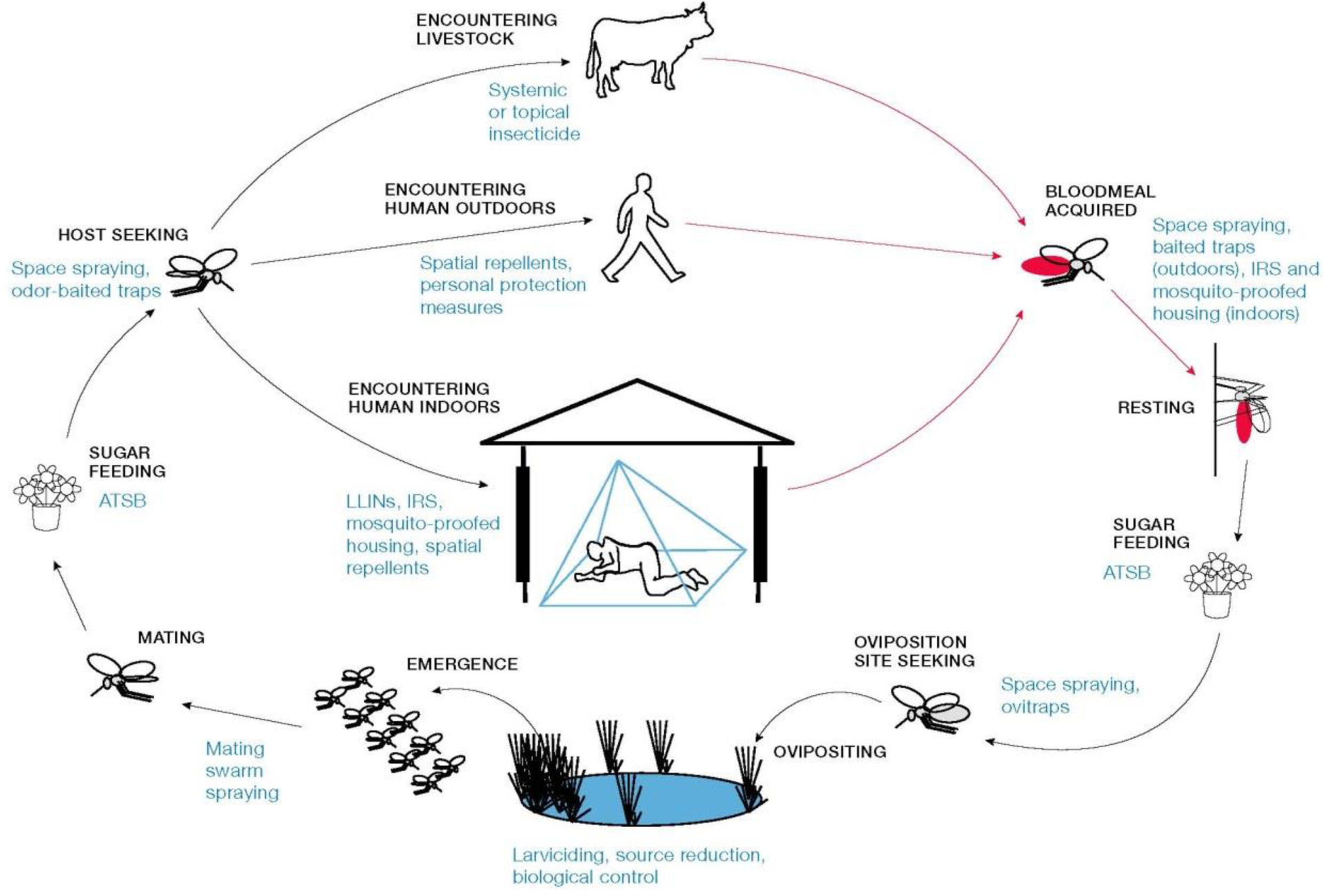

Supplement: S2 Fig — The schematic highlights opportunities for vector control to target biological and environmental mosquito resources. (TIF) [file pone.0187680.s002.tif]

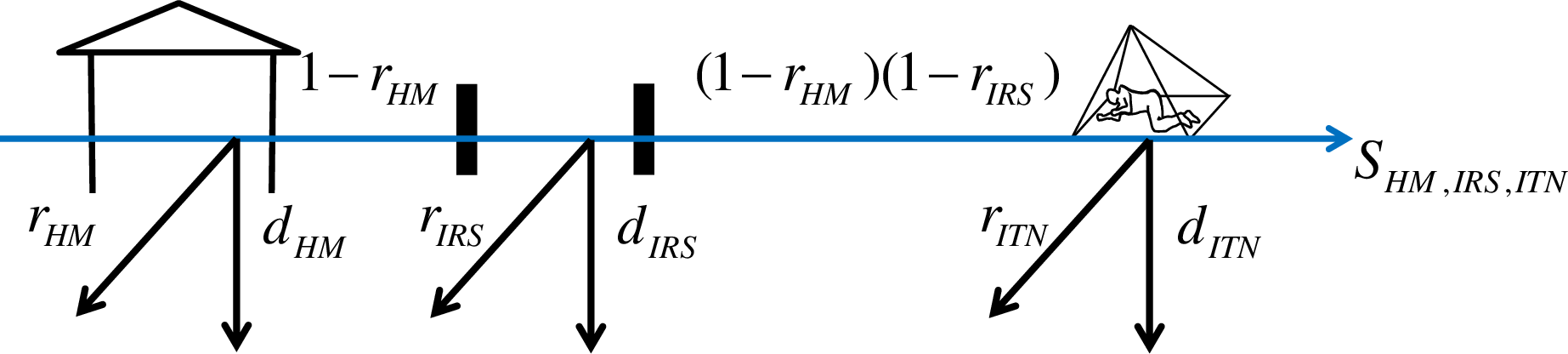

Supplement: S3 Fig — The schematic illustrates the probability of mosquito being repelled or killed upon encountering indoor interventions. (TIF) [file pone.0187680.s003.tif]

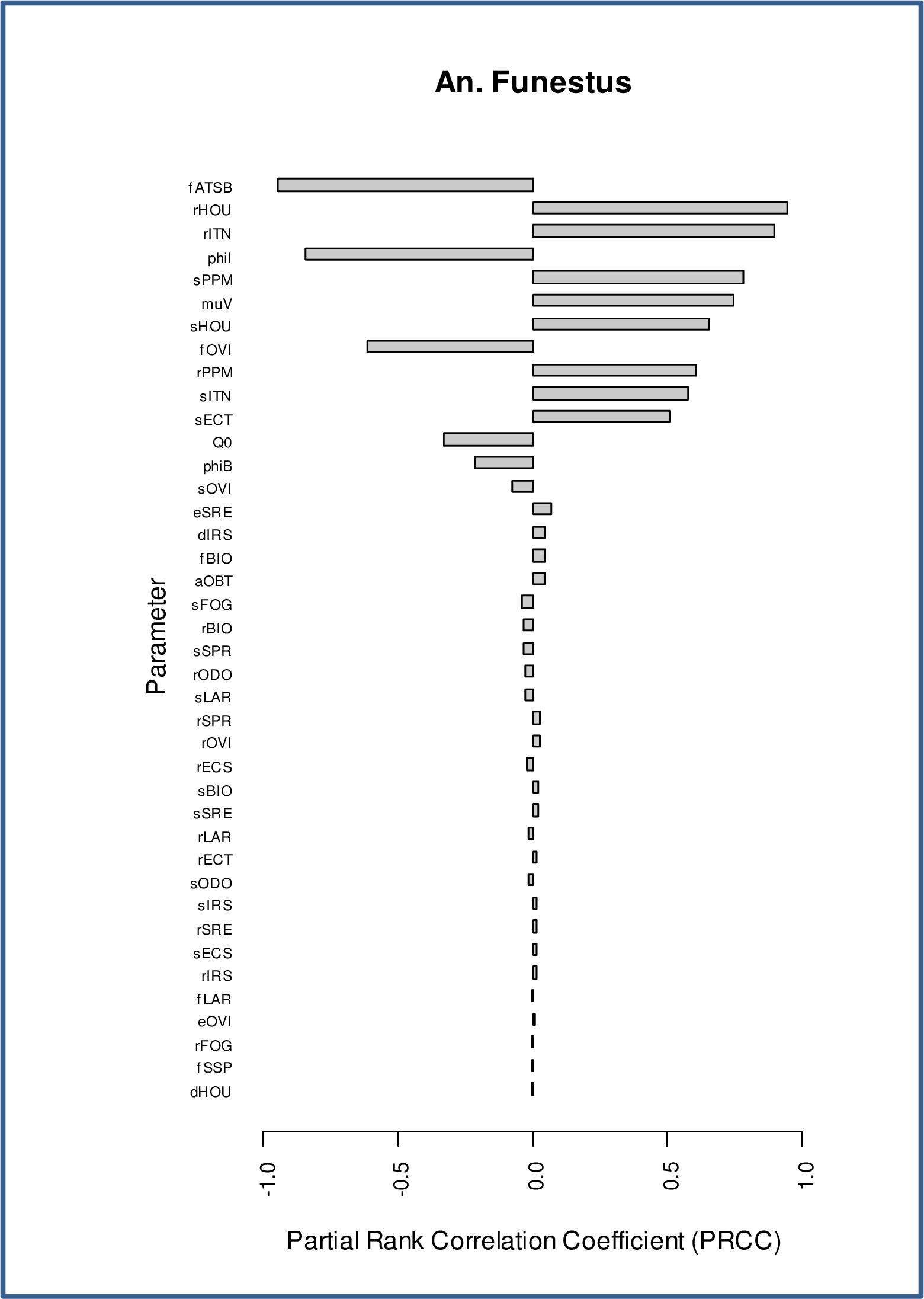

Supplement: S4 Fig — (TIF) [file pone.0187680.s004.tif]

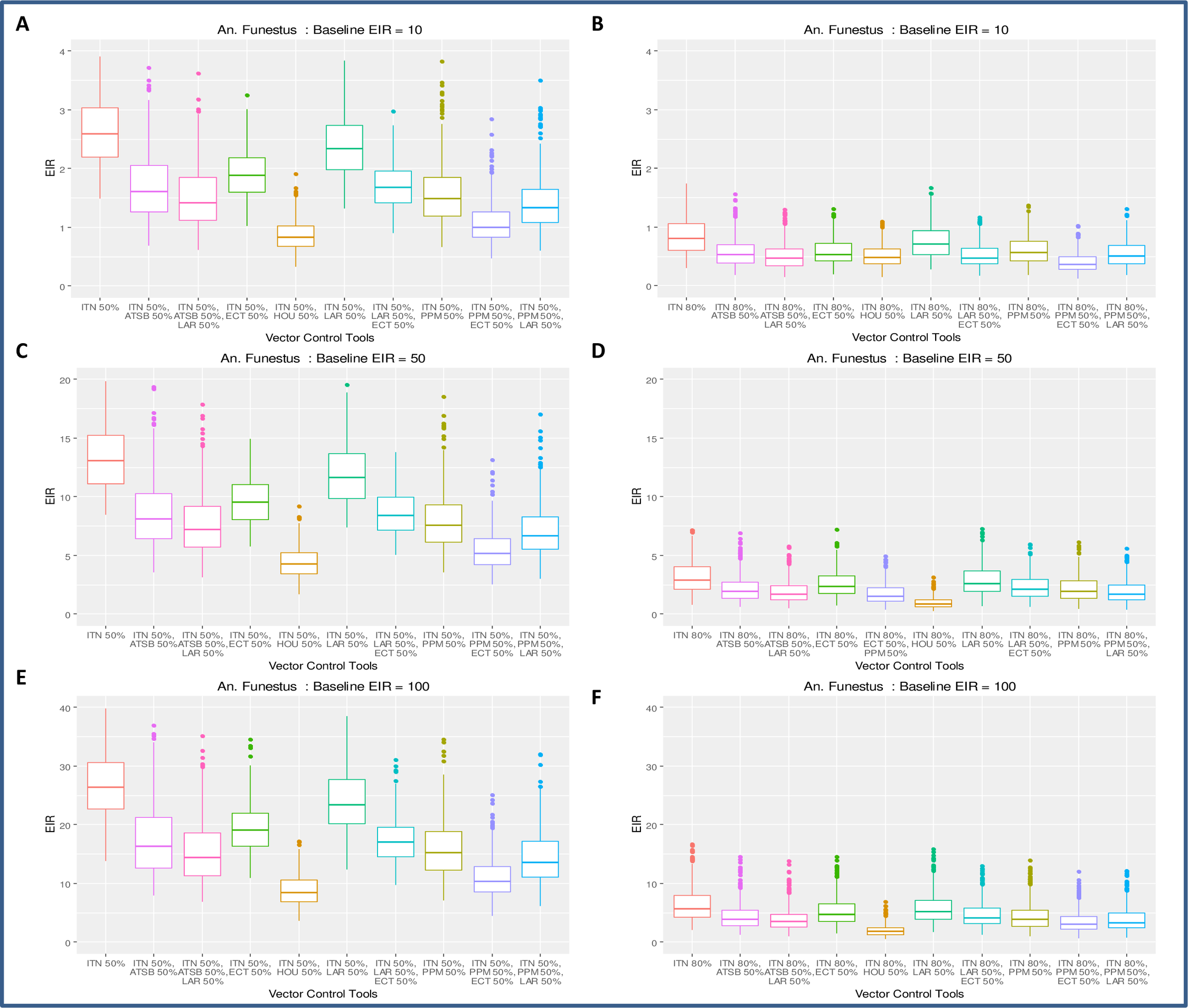

Supplement: S5 Fig — (TIF) [file pone.0187680.s005.tif]

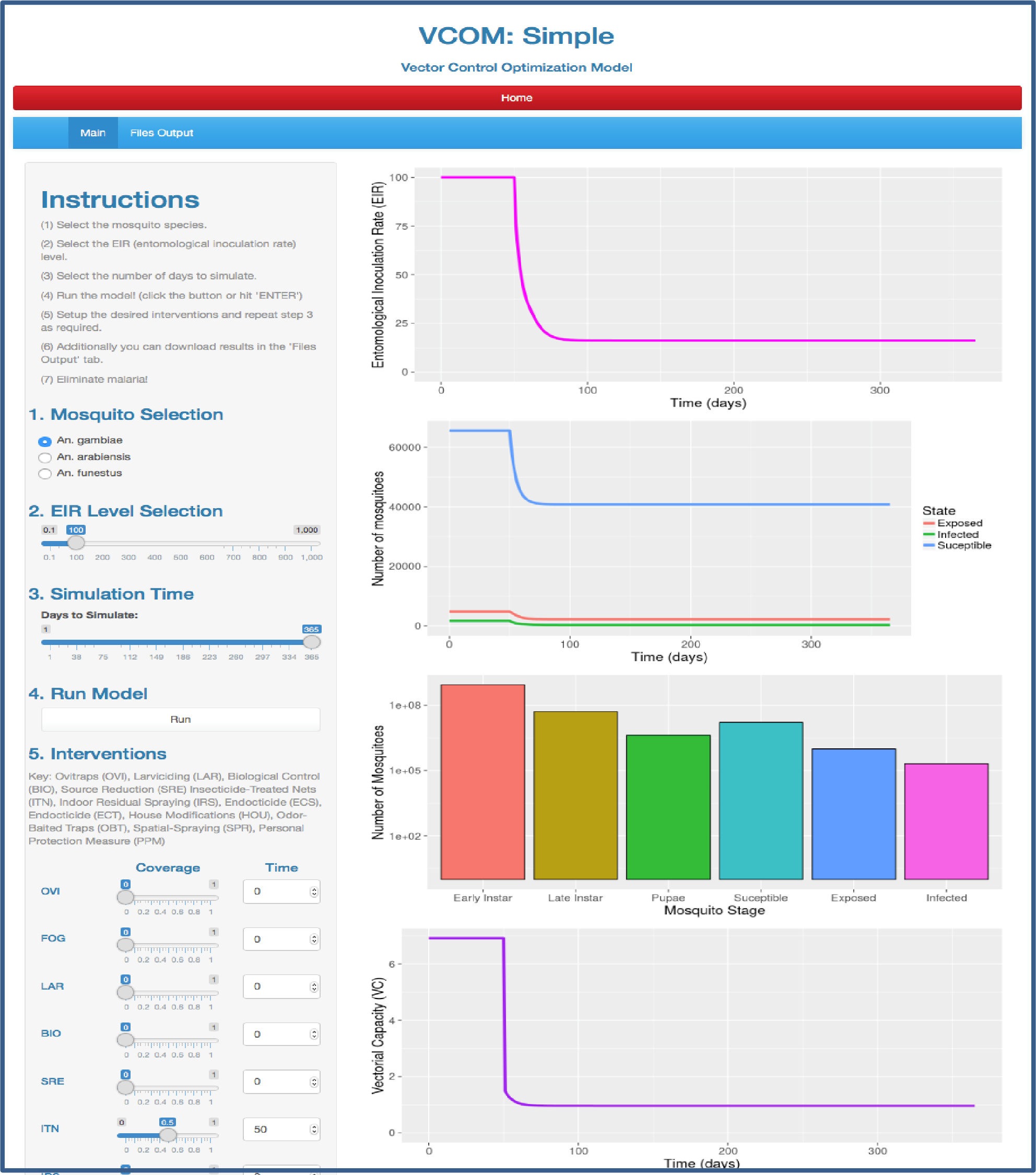

Supplement: S6 Fig — (TIF) [file pone.0187680.s006.tif]
