# Supplementary material for: Attacking the mosquito on multiple fronts: Insights from the Vector Control Optimization Model (VCOM) for malaria elimination
Source: PLoS One. 2017 Dec 1;12(12):e0187680. doi: 10.1371/journal.pone.0187680 (PMC5711017; doi:10.1371/journal.pone.0187680)
Supplement: S1 Table — (DOCX) [file pone.0187680.s007.docx]

| **Parameter:** | **Description:** | **Value:** | **Reference:** |
| --- | --- | --- | --- |
|  | Average duration of infectiousness in untreated humans | 50 days | [1] |
|  | Average mosquito life expectancy | 8 days | [2, 3] |
| $\tau$ | Average latent period in mosquito host | 10 days | [4] |
|  | Biting rate per mosquito on humans | 0.33 per day | [5] |
|  | Vector-to-human transmission probability | 0.2 | [6-8] |
|  | Human-to-vector transmission probability | 0.05 | [9, 10] |
| $\beta$ | Number of eggs laid per adult female mosquito per day | 21.19 | [11] |
| *$d_{E}$* | Developmental period of early instars | 6.64 days | [12] |
| $\mu_{E}$ | Death rate of early instars | 0.034 per day | [12] |
| $d_{l}$ | Developmental period of late instars | 3.72 days | [12] |
| $\mu_{L}$ | Death rate of late instars | 0.035 per day | [12] |
| $\gamma$ | Density-dependent factor for late instars | 13.25 | [11] |
| $d_{P}$ | Developmental period of pupae | 0.64 days | [12] |
| $\mu_{P}$ | Death rate of pupae | 0.25 per day | [11] |
| $1/f(0)$ | Reciprocal of gonotrophic cycle length | 0.33 per day | [13] |
| $t_{1}$ | Mean time spent foraging for a blood-meal | 0.68 days | [14] |
| $t_{2}$ | Mean time spent resting and ovipositing | 2.32 days | [14] |
