# Supplementary material for: Attacking the mosquito on multiple fronts: Insights from the Vector Control Optimization Model (VCOM) for malaria elimination
Source: PLoS One. 2017 Dec 1;12(12):e0187680. doi: 10.1371/journal.pone.0187680 (PMC5711017; doi:10.1371/journal.pone.0187680)
Supplement: S2 Table — (DOCX) [file pone.0187680.s008.docx]

| **Parameter:** | **Definition:** | ***An. gambiae*:** | ***An. arabiensis*:** | ***An. funestus:*** | **Reference:** |
| --- | --- | --- | --- | --- | --- |
| $Q_{h,0}$ | Human blood index – baseline proportion of blood-meals obtained from human by a mosquito | 0.92 | 0.71 | 0.94 | [1, 2] |
| $1/\mu_{V}$ | Adult mosquito life expectancy | 7.6 | 7.6 | 8.9 | [3-5] |
|  | Proportion of bites on a person in bed | 0.89 | 0.90 | 0.90 | [6] |
|  | Proportion of bites on a person indoors | 0.97 | 0.96 | 0.98 | [6, 7] |
