# Supplementary material for: Attacking the mosquito on multiple fronts: Insights from the Vector Control Optimization Model (VCOM) for malaria elimination
Source: PLoS One. 2017 Dec 1;12(12):e0187680. doi: 10.1371/journal.pone.0187680 (PMC5711017; doi:10.1371/journal.pone.0187680)
Supplement: S3 Table — (DOCX) [file pone.0187680.s009.docx]

| **Parameter:** | **Definition:** | ***An. gambiae*:** | ***An. arabiensis*:** | ***An. funestus:*** | **Reference:** |
| --- | --- | --- | --- | --- | --- |
| $r_{ITN}$ | Probability of repeating a feeding attempt due to ITNs | 0.56  (0.5 – 0.7) | 0.48  (0.5 – 0.7) | 0.56  (0.5 – 0.7) | [1] |
| $S_{ITN}$ | Probability of feeding and surviving in presence of ITNs | 0.03  (0.03-0.1) | 0.39  (0.03-0.1) | 0.03  (0.03-0.1) | [1, 2] |
| $r_{IRS}$ | Probability of repeating a feeding attempt due to IRS | 0.60  (0.5 – 0.7) | 0.60  (0.5 – 0.7) | 0.63  (0.5 – 0.7) | [3] |
| $S_{IRS}$ | Probability of feeding and surviving in presence of IRS | 0  (0.02 - 0.1) | 0  (0.02 - 0.1) | 0  (0.02 - 0.1) | [3] |
| $d_{IRS}$ | Mosquito death rate due to IRS | 0.40  (0.35 – 0.5) | 0.40  (0.35 – 0.5) | 0.37  (0.35 – 0.5) | [4] |
| $d_{ITN}$ | Mosquito death rate due to ITNs | 0.41  (0.35 – 0.5) | 0.13  (0.1 – 0.2) | 0.41  (0.35 – 0.5) | [1, 2] |
| $d_{HM}$ | Mosquito death rate due to mosquito proofed housing | 0.1  (0.05 – 0.2) | 0.1  (0.05 – 0.2) | 0.1  (0.05 – 0.2) | Assumed/Varied |
| $r_{HM}$ | Probability of being repelled during a feeding attempt due mosquito proofed housing | 0.56  (0.5 – 0.7) | 0.48  (0.5 – 0.7) | 0.56  (0.5 – 0.7) | Assumed/Varied |
| $S_{HM}$ | Probability of feeding and surviving in presence due to mosquito proofed housing | 0.03  (0.02 – 0.1) | 0.39  (0.02 – 0.1) | 0.03  (0.02 – 0.1) | Assumed/Varied |
| $r_{SR}$ | Probability of being repelled during a feeding attempt due to spatial repellents | 0.82  (0.7 - 0.82) | (0.82) *  (0.7 - 0.82) | (0.82) *  (0.7 - 0.82) | [5] |
| $S_{SR}$ | Probability of feeding and surviving in presence of spatial repellents | 0.12  (0.12 – 0.3) | 0.02  (0.12 – 0.3) | (0.12) *  (0.12 – 0.3) | [5]) |
| $r_{PPM}$ | Probability of being repelled during a feeding attempt due to personal protection measures | 0.69  (0.5 – 0.7) | 0.69  (0.5 – 0.7) | 0.69  (0.5 – 0.7) | Assumed/Varied |
| $S_{PPM}$ | Probability of feeding and surviving in presence of personal protection measure | 0.31  (0.2 – 0.4) | (0.31) *  (0.2 – 0.4) | (0.31) *  (0.2 – 0.4) | [6] |
| $r_{ct}$ | Probability of being repelled during a feeding attempt due to topical insecticide treated cattle | 0.4  (0.2 – 0.5) | 0.4  (0.2 – 0.5) | 0.4  (0.2 – 0.5) | Assumed/Varied |
| $S_{ct}$ | Probability of feeding and surviving in presence of topical insecticide treated cattle | 0.5  (0.4 - 0.6) | 0.5  (0.4 - 0.6) | 0.5  (0.4 - 0.6) | Assumed/Varied |
| $r_{cs}$ | Probability of being repelled during a feeding attempt due to systemic insecticide treated cattle | 0.2  (0.2 – 0.8) | 0.2  (0.2 – 0.8) | 0.2  (0.2 – 0.8) | Assumed/Varied |
| $S_{cs}$ | Probability of feeding and surviving in presence of systemic insecticide treated cattle | 0.10  (0.1 – 0.5) | 0  (0.1 – 0.5) | 0.10  (0.1 – 0.5) | [7] |
| $\zeta_{ATSB}$ | Factor allowing for mosquito increased death due to ATSB | 17.1  (15 – 20) | 17.1  (15 – 20) | 11.7  (10 – 15) | [8] |
| $\zeta_{SS}$ | Factor allowing for mosquito increased death due to space spraying | 1.5  (1- 2) | 1.5  (1- 2) | 1.5  (1- 2) | Assumed/Varied |
| $\zeta_{OT}$ | Factor allowing for mosquito increased death due to ovitraps | 1.5  (1- 2) | 1.5  (1- 2) | 1.5  (1- 2) | Assumed/Varied |
| $e_{K}$ | Effectiveness of the impact of source reduction in environmental carrying capacity (K) | 0.5  (0.35 – 0.50) | 0.5  (0.35 – 0.50) | 0.5  (0.35 – 0.50) | [9] |
| $e_{OT}$ | Effectiveness of the ovitraps | 0.5  (0 – 1) | 0.5  (0 – 1) | 0.5  (0 – 1) | Assumed/Varied |
| $\zeta_{l}$ | Factor allowing for mosquito increased death rate due to larviciding | 55.2  (50 – 60) | 55.2  (50 – 60) | 55.2  (50 – 60) | [10] |
| $\zeta_{bc}$ | Factor allowing for mosquito increased death rate due to biological control | 38.1  (35 – 50) | 38.1  (35 – 50) | 38.1  (35 – 50) | [11] |
| $\alpha_{t}$ | Availability of one odor-baited trap in relation to one human | 2  (1 – 4) | 2  (1 – 4) | 2  (1 – 4) | Assumed/Varied |

*Data not obtained but are assumed to be same as for the other specie where data is cited.

Range of parameter values used for sensitivity analysis is given in brackets

Parameters values are assumed with values varied where data is not available
