# Supplementary material for: Attacking the mosquito on multiple fronts: Insights from the Vector Control Optimization Model (VCOM) for malaria elimination
Source: PLoS One. 2017 Dec 1;12(12):e0187680. doi: 10.1371/journal.pone.0187680 (PMC5711017; doi:10.1371/journal.pone.0187680)
Supplement: S1 Appendix — (DOCX) [file pone.0187680.s010.docx]

**Attacking the mosquito on multiple fronts: Insights from the Vector Control Optimization Model (VCOM) for malaria elimination**

Samson S. Kiware­^1,2^, Nakul Chitnis^3,4^, Allison Tatarsky^5^, Sean Wu^6^, Héctor Manuel Sánchez Castellanos^6,7^, Roly Gosling^5^, David Smith^8^, John Marshall^6^

1. *Biomedical and Environmental Thematic Group, Ifakara Health Institute, Morogoro,Tanzania*
2. *Mathematics, Statistics, and Computer Science Department, Marquette University, Milwaukee, WI, USA*
3. *Department of Epidemiology and Public Health, Swiss Tropical and Public Health Institute, Basel, Switzerland*
4. *University of Basel, 4003 Basel, Switzerland*
5. *Malaria Elimination Initiative, Global Health Group, University of California, San Francisco, USA*
6. *Divisions of Biostatistics and Epidemiology, University of California, Berkeley*
7. *School of Medicine, Tecnologico de Monterrey, Atizapan de Zaragoza, Estado de Mexico, 64849,*
8. *Mexico Department of Global Health, University of Washington, Seattle, USA*

# S1 Appendix

In this document, we present detailed mathematical equations of the mosquito ecological model, the mosquito Susceptible – Exposed - Infected (SEI) model, and the human Susceptible - Infected model, as presented in Fig.A.

**Fig A. Model schematic diagram.** Flow diagram for the mosquito ecological model, mosquito SEI model, and human SI model.

Mosquito ecological model

Here, we present the compartment model describing mosquito ecology (i.e. progress from egg to larva to pupa to adult). The mosquito ecological model has been previously described in detail by White *et al.* (2011). In summary: 1) each female mosquito lays on average $\beta$ eggs per day that hatch into early-instar larvae, $E$; 2) early instar larvae are subject to density-dependent daily mortality at a rate $\mu_{E}$ and, if they survive, progress to late-instar larvae, $L$, after $d_{E}$ days ; 3) Late-instar larvae undergo density-dependent daily mortality at a rate $\mu_{L}$ and, if they survive, develop into pupae $(P)$ after $d_{L}$ days; and 4) pupae undergo density-independent mortality at a rate $\mu_{P}$ or progress into adult mosquitoes (where half are assumed to be female), $V$, after $d_{P}$ days.

The mosquito life cycle can thus be described by the following set of continuous ordinary differential equations:

$\frac{dE}{dt}=\beta V-\mu_{E}\left(1+\frac{E+L}{K}\right)E-\frac{E}{d_{E}}$ , (S1. 1)

$\frac{dL}{dt}=\frac{E}{d_{E}}-\mu_{L}\left(1+\gamma\frac{E+L}{K}\right)L-\frac{L}{d_{L}}$ , (S1. 2)

$\frac{dP}{dt}=\frac{L}{d_{L}}-\mu_{P}P-\frac{P}{d_{P}}$ , (S1. 3)

$\frac{dV}{dt}=\frac{1}{2}\frac{P}{d_{P}}-\overline{\mu_{V,0}}V$ , (S1. 4) $\frac{dM_{m}}{dt}=\frac{1}{2}\frac{P}{d_{P}}-\mu_{M}M_{m}$ (S1. 5)

Here, $V$ represents the number of adult female mosquitoes, $K$ is the carrying capacity of the environment for larvae, and $\gamma$ is a factor allowing for the differential effects of density-dependent mortality on early and late-instar larvae. $\overline{\mu_{V,0}}$ represents the daily average mortality rate that adult female mosquitoes undergo in the absence of interventions, $M_{m}$ represent the number of male adult mosquitoes, and$\mu_{M}$ represents the daily mortality rate that adult male mosquitoes undergo.

At equilibrium, $V_{eq}$ [1] can then be given by;

$V_{eq}=\frac{K\left[\left(\frac{\omega}{\mu_{L}d_{E}}\right)-\left(\frac{1}{\mu_{L}d_{L}}\right)-1\right]}{2\overline{\mu_{V,0}}d_{L}(1+\mu_{P}d_{P})\gamma(\omega+1)}$ (S1. 6)

For every$V_{eq}$, assuming other parameters are fixed, there is a unique value of $K$ that gives you that value of $V_{eq}$. Where, $\omega$ is a function of mosquito ecological parameters given by [1];

$\omega=\frac{-\beta_{\omega}}{2}+\sqrt{\frac{\beta_{\omega}^{2}}{4}+\frac{\gamma\beta\mu_{L}d_{E}}{2\mu_{E}\overline{\mu_{V,0}}d_{L}(1+\mu_{P}d_{P})}}$ (S1. 7)

and $\beta_{\omega}$[1] is given by,

$\beta_{\omega}=\gamma\frac{\mu_{L}}{\mu_{E}}-\frac{d_{E}}{d_{L}}+(\gamma-1)\mu_{L}d_{E}$ (S1. 8)

# Malaria Dynamics

Shortly, we will elaborate on the feeding cycle model, which describes the factors contributing to the rate at which female mosquitoes feed and lay eggs. Initially, however, we will expand upon Equations S1. 1-4 by introducing malaria infection into the mosquito population. Equation S1. 4 describes the rate of change in the total female mosquito population size. We now subdivide the adult female mosquito population into those susceptible, latently infected and infectious for the malaria parasite, *S_V_*, *E_V_* and *I_V_*, respectively. The equation for susceptible vectors is now given by,

$\frac{dS_{V}}{dt}=\frac{1}{2}\frac{P}{d_{P}}-a \left(\frac{I_{H}}{H}\right)b_{V}S_{V}-\overline{\mu_{V,0}}S_{V}$ (S1. 9)

Here, *a* represents that rate at which a given adult female mosquito bites humans, *I_H_*/*H* represents the fraction of the human population that is infectious for malaria parasites, and *b_V_* represents proportion of bites on infectious humans that result in a susceptible mosquito becoming infected with the parasite.

After completing a latent infection period, $\tau$, a vector becomes infectious ($I_{V}$). Considering a fixed latent period, a fraction df of mosquitoes survive this latent period to become infectious. The flow of mosquitoes entering the infectious compartment can therefore be denoted by a delayed differential equation as,

$\frac{dI_{V}}{dt}=a \left(\frac{I_{H}(t-\tau)}{H}\right)b_{V}S_{V}(t-\tau)e^{-\overline{\mu_{V}}\tau}-\overline{\mu_{V,0}}I_{V}$ (S1. 10)

Here, $\tau$ represents the duration of the latent period, $I_{H}(t-\tau)$ represents the number of infectious humans $\tau$days prior to time *t*, $S_{V}(t-\tau)$ represents the number of susceptible vectors$\tau$days prior to time $t$, and $e^{-\overline{\mu}_{V}\tau}$ represents the proportion of vectors that survive the latent period.

The remainder of the adult female mosquito population is latently infected. So, given the force of infection on vectors is $\lambda_{V}=a \left(\frac{I_{H}}{H}\right)b_{V}$and noting the flows of mosquitoes into the infectious compartment and out of the susceptible compartment, the latent vector stage $(E_{V})$ can be described by,

$\frac{dE_{V}}{dt}=a \left(\frac{I_{H}}{H}\right)b_{V}S_{V}-a \left(\frac{I_{H}(t-\tau)}{H}\right)b_{V}S_{V}(t-\tau)e^{-\overline{\mu_{V,0}}\tau}-\overline{\mu_{V,0}}E_{V}$ (S1. 11)

So, the total vectors is given by $V=S_{V}+E_{V}+I_{V}.$

As we are focusing on the mosquito population dynamics, we describe malaria infection in the human population using a simple susceptible-infectious (SI) model - a susceptible human ($S_{H}$) becomes infected ($I_{H}$ ) when bitten by a mosquito at a human biting rate, *a*, with a proportion of bites producing infection in humans depending on proportion of mosquitoes that are infectious for the parasite, $\frac{I_{V}}{V}$, and the probability of human infection given an infectious mosquito bites a susceptible human, *b_V_*. The number of mosquitoes per human, *V*/*H*, is also accounted for. Infected humans are assumed to recover from infection at a rate, $r$. Thus, given the force of infection in humans is $\lambda_{H}=\left(\frac{V}{H}\right)a \left(\frac{I_{V}}{V}\right)b_{H}$, then, the differential equations describing these dynamics are:

$\frac{dS_{H}}{dt}=-\left(\frac{V}{H}\right)a \left(\frac{I_{V}}{V}\right)b_{H}S_{H}+rI_{H}$ (S1. 12)

$\frac{dI_{H}}{dt}=\left(\frac{V}{H}\right)a \left(\frac{I_{V}}{V}\right)b_{H}S_{H}-rI_{H}$ (S1. 13)

Where, $H=S_{H}+I_{H}$ and $a$ is the human feeding rate given by $a=Q_{0}/f(0)$ and $f(0)$ is defined and described in the next subsection.

We can now present equations describing the female mosquito feeding cycle in the absence of interventions.

Female mosquito feeding cycle in the absence of interventions

We first provide equations [2] describing the mosquito population parameters in absence of interventions.

### The time to complete one feeding cycle

After emergence, female mosquitoes seek humans for blood-feeding. This process is assumed to take $t_{1}(0)$ days. Following a successfully feed, a mosquito rests, find larval habitat and oviposits eggs. This process is assumed to take $t_{2}(0)$ days. Thus, the average time to complete one feeding cycle, $f(0)$, in the absence of an intervention is given by,

$f\left( 0 \right)=t_{1}\left( 0 \right)+t_{2}(0)$ (S1. 14)

### Probability of a mosquito surviving one day in the absence of interventions

The probability of a mosquito surviving foraging in the absence of any intervention is equal to:

$p_{1}(0)=e^{-\mu_{V,1}t_{1}(0)}$ . (S1. 15)

Here, $\mu_{V,1}$ represents the mosquito death rate during foraging. The probability of a mosquito surviving resting and ovipositing is given by:

$p_{2}(0)=e^{-\mu_{V,2}t_{2}}$ (S1. 16)

Here, $\mu_{V,2}$ represents the mosquito death rate during resting and ovipositing. Thus, the probability $p\left( 0 \right)$ of surviving one day in the absence of an intervention is given by:

$P(0)=\left[p_{1}(0)p_{2}(0)\right]^{1/f(0)}$ (S1. 17)

Here, we have assumed that the death rate during foraging is not the same as death rate during resting and ovipositing. This will allow us to alter the stage-specific death rates according to the interventions being used as well.

### Computing female mosquito death rate in absence of interventions

The mean mosquito death rate can be computed as follows:

$\overline{\mu_{V,0}}=-\log\left(P(0)\right)$ (S1. 18)

### Number of eggs per oviposition per mosquito

In the absence of vector control interventions, the rate at which female mosquitoes oviposit eggs,$\beta_{0}$, (for the normal adult male to female mosquito ratio) is given by [1],

$\beta_{0}=\frac{\varepsilon\overline{\mu_{v,0}}}{e^{\overline{\mu_{V,0}}f(0)}-1}$ . (S1. 19)

Here, *ε* is the number of viable eggs that a female mosquito lays per oviposition cycle, and the other terms have already been defined.

The impact of interventions - targeting mosquitoes when using their resources

The impact of interventions is modeled by the effects they have on diverting mosquitoes - which can increase the amount of time it takes for the gonotrophic cycle to be completed, and hence decreases the mosquito feeding/biting rate.

**Fig B. Targeting mosquito on multiple fronts:** The schematic highlights opportunities for vector control to target biological and environmental mosquito resources.

Other effect which can be modeled are the effect of interventions on the adult mosquito daily mortality rate and the impact in killing all immature mosquito forms in aquatic habitats, which impacts the total mosquito population size, and the effect of these interventions on reducing the human biting rate, which is relevant for malaria transmission from both mosquito-to-human and human-to-mosquito. We therefore describe different vector control interventions that can be used to target mosquito while utilizing a given resource (see Fig B).

## **Targeting mosquito in aquatic habitats**

We consider three interventions that can be used to target the immature mosquito forms and eventually reduce the mosquito emergence rate. These are source reduction, larvaciding and biological control.

### The impact of source management in reducing environmental carrying-capacity

We can incorporate the impact of source reduction in the model by considering its impact on environmental carrying capacity (*K*) as follows;

$K_{\theta}=(1-e_{K} C_{K})K$ (S1. 20)

Here, $e_{K}$ is the effectiveness (0 to 1) of the impact of source reduction in K, and $C_{K}$ is the proportion of aquatic habitats where source reduction is implemented as an intervention.

We can then substitute $K$ with $K_{\theta}$ in Equations S1 1-2 to capture the impact of source reduction on the early and late-instar life stages, i.e.:

$\frac{d}{dt}(E)=\beta V-\mu_{E}\left(1+\frac{E+L}{K_{\theta}}\right)E-\frac{E}{d_{E}}$ (S1. 21)

$\frac{d}{dt}(L)=\frac{E}{d_{E}}-\mu_{L}\left(1+\gamma\frac{E+L}{K_{\theta}}\right)L-\frac{L}{d_{L}}$ (S1. 22)

### The impact of larvaciding and biological control in suppressing the immature mosquito population

Let’s denote larvacide coverage by $\chi_{l}$ which we interpret as the proportion of aquatic habitats covered by larvacide, and biological control coverage by $\chi_{bc}$ which we interpret as the proportion of aquatic habitats covered by biological control. Thus, the coverage parameters, $C_{l}$, $C_{bc}$, $C_{l,bc}$ and $C_{l,bc,0}$represent coverage with larvacide only, biological control only, larvacide and biological control, and with neither larvacide or biological control respectively. Assuming independent coverage of each intervention, then,

$C_{l}=\chi_{l}-\chi_{l}\chi_{bc}$ , (S1. 23)

$C_{bc}=\chi_{bc}-\chi_{l}\chi_{bc}$ , (S1. 24)

$C_{l,bc}=\chi_{l}\chi_{bc}$ , (S1. 25)

$C_{l,bc,0}=1-\chi_{l}-\chi_{bc}+\chi_{l}\chi_{bc}$ (S1. 26)

If we let $\zeta_{l}$ and $\zeta_{bc}$be factors allowing for increased death rate due to larvacide and biological control respectively. Assuming the two interventions act independently to each other, then increased mosquito death rate due to larvacide and biological control is given by:

$\zeta_{l,bc}=\zeta_{l}\zeta_{bc}$ (S1. 27)

Now, in order to incorporate larvaciding and biological control in the model - we need to modify Equations S1. 21 and S1. 22 by including categories $E_{l}$, $E_{bc}$, $E_{l,bc}$, $L_{l}$, $L_{bc}$, $L_{l,bc}$, $P_{l}$, $P_{bc}$ and $P_{l,bc}$ to represent early instars, late instars, and pupae, respectively, which reside in aquatic habitats where these interventions are applied alone and/or in combination, thus;

$\frac{dE}{dt}=C_{l,bc,0}\beta_{\theta}V-\mu_{E}\left(1+\frac{E+L}{C_{l,bc,0}K_{\theta}}\right)E-\frac{E}{d_{E}}$ , (S1. 28)

$\frac{dE_{l}}{dt}=C_{l}\beta_{\theta}M-\zeta_{l}\mu_{E}\left(1+\frac{E_{l}+L_{l}}{C_{l}K_{\theta}}\right)E_{l}-\frac{E_{l}}{d_{E}},$ (S1. 29)

$\frac{dE_{bc}}{dt}=C_{bc}\beta_{\theta}M-\zeta_{bc}\mu_{E}\left(1+\frac{E_{bc}+L_{bc}}{C_{bc}K_{\theta}}\right)E_{bc}-\frac{E_{bc}}{d_{E}},$ (S1. 30)

$\frac{dE_{l,bc}}{dt}=C_{l,bc}\beta_{\theta}M-\zeta_{l,bc}\mu_{E}\left(1+\frac{E_{l,bc}+L_{l,bc}}{C_{l,bc}K_{\theta}}\right)E_{l,bc}-\frac{E_{l,bc}}{d_{E}},$ (S1. 31)

$\frac{dL}{dt}=\frac{E}{d_{E}}-\mu_{L}\left(1+\gamma\frac{E+L}{C_{l,bc,0}K_{\theta}}\right)L-\frac{L}{d_{L}}$ , (S1. 32)

$\frac{dL_{l}}{dt}=\frac{E_{l}}{d_{E}}-\zeta_{l}\mu_{L}\left(1+\gamma\frac{E_{l}+L_{l}}{C_{l}K_{\theta}}\right)L_{l}-\frac{L_{l}}{d_{L}}$ (S1. 33)

$\frac{dL_{bc}}{dt}=\frac{E_{bc}}{d_{E}}-\zeta_{bc}\mu_{L}\left(1+\gamma\frac{E_{bc}+L_{bc}}{C_{bc}K_{\theta}}\right)L_{bc}-\frac{L_{bc}}{d_{L}}$ (S1. 34)

$\frac{dL_{l,bc}}{dt}=\frac{E_{l,bc}}{d_{E}}-\zeta_{l,bc}\mu_{L}\left(1+\gamma\frac{E_{l,bc}+L_{l,bc}}{C_{l,bc}K_{\theta}}\right)L_{l,bc}-\frac{L_{l,bc}}{d_{L}}$ (S1. 35)

It is assumed that the proportion of eggs mosquitoes lay in breeding sites correspond to its carrying capacity. The value of any of the given coverage (C) in equations is greater than 0, otherwise, the equation where C is 0 is excluded in the system. It is expected that the impact of aerial larvaciding will be higher as compared to conventional larviciding since it is expected that high coverage of aquatic habitats $\left(C_{l}\right)$with larvacide can be reached with aerial larvaciding.

## Targeting mosquitoes while mating

After emergence, female mosquitoes mate in flight with male mosquitoes which normally gather in swarms at specific mating sites over landmarks (Diabate & Tripet, 2015). We can target mosquito while mating by increasing male mosquito death rate $\left(\mu_{M}\right)$ by using swarm spraying intervention as follows;

$\mu_{M,\theta}=\mu_{M}\zeta_{SwS}.$ (S1. 36)

The egg-laying rate of adult females,$\beta_{0}$ can now be denoted by$\beta(M_{m},V)$, as a function of the ratio of adult male mosquitoes to adult female mosquitoes, $M_{m}/V$ . As the number of adult male mosquitoes decreases, it becomes more difficult for adult female mosquitoes to find a mate, and hence the egg-laying rate of adult females decreases. The following is a sigmoidal function that may be used to describe this relationship;

$\beta_{\theta,mss}=\beta_{0}\left(1-\frac{1}{1+e^{-\alpha((M_{m}/V)-\upsilon)}}\right)$ . (S1. 37)

Here, $\beta_{0}$ represents the egg-laying rate for the normal adult gender ratio (Equation S1. 19 ), *υ* represents the critical gender ratio at which the egg-laying rate is half that of the normal rate, and *α* is a parameter representing the rate at which the egg-laying rate decreases within the vicinity of the critical gender ratio. The values of *α* and *υ* are estimated using the data obtained from a study performed in Burkina Faso on male swarm spraying (Diabate, Unpublished)

Here, we have ignored the fact that female mosquitoes mate only once in the life time but can lay eggs more than once.

## **Targeting mosquitoes while searching for blood**

Mosquitoes can be targeted while host seeking by using space spraying and/or using baited traps. These interventions can both increase the mosquito death rate.

### Estimating the impact of attractive toxic sugar baits and space spraying on host-seeking mosquitoes

Recall that, if $\mu_{v,1}$ represents the natural death rate of adult female mosquitoes during host-seeking and after emergence and a mosquito host-seeking process takes $t_{1}(0)$ days, then the probability of surviving this process, $p_{1}$, in the absence of an intervention is given by $p_{1}=\exp(-\mu_{v,1}t_{1}(0))$. Here, we can update $p_{1}$ to incorporate the impact of intervention by considering the fact that attractive toxic sugar baits (ATSB) and space spraying (SS) increase the mosquito death rate, $\mu_{v,1}$.

Let’s denote ATSB coverage by $\chi_{ATSB}$, which we interpret as the proportion of vegetation sprayed with or outdoor baited stations provided with ATSB, and space spraying coverage by $\chi_{SS}$, which we interpret as the proportion of space sprayed with insecticide in a specific defined area. Thus, the coverage parameters, $C_{ATSB}$, $C_{SS}$, $C_{ATSB,SS}$, and $C_{ATSB,SS,0}$ represent coverage with ATSB only, SS only, ATSB and SS, and with neither ATSB or SS respectively. Assuming independent coverage of each intervention, then,

$c_{ATSB}=\chi_{ATSB}-\chi_{ATSB}\chi_{SS},$ (S1. 38)

$c_{SS}=\chi_{SS}-\chi_{ATSB}\chi_{SS},$ (S1. 39)

$c_{ATSB,SS}=\chi_{ATSB}\chi_{SS},$ (S1. 40)

$c_{ATSB,SS,0}=1-\chi_{ATSB}-\chi_{SS}+\chi_{ATSB}\chi_{SS}.$ (S1. 41)

If we let $\zeta_{ATSB}$ and $\zeta_{SS}$ be the factor allowing for increased $\mu_{v,1}$ in presence of ATSB and SS respectively, then the death rate due ATBS and SS is be given by:

$\mu_{v,1,\theta}= \mu_{v,1}\left[C_{ATSB}\zeta_{ATSB}+C_{SS}\zeta_{SS}+C_{ATSB,SS}\zeta_{ATSB}\zeta_{SS}+C_{ATSB,SS,0}\right]$ (S1. 42)

Thus, at this stage, the probability of surviving host-seeking process in the presence of these interventions can be given by:

$p_{1,\theta}=e^{-\mu_{V,1,\theta}t_{1}}$ (S1. 43)

### Estimating population level effects of odor-baited traps

The efficacy of odor-bated traps is derived primarily from two complementary characteristics: 1) their high attractiveness to mosquitoes compared to attractiveness of humans; and 2) their ability to trap and kill mosquitoes which attack them thus removing these mosquitoes from the biting population.

We let $\alpha_{t}$denote the availability of one trap in relation to one human and $r_{t}$ to represent a ratio of traps to humans (similar to proportion of trap coverage). Considering that mosquitoes attempt to feed when attacking odor-baited traps –we can incorporate the impact of traps by its ability to trap and kills mosquitoes hence reducing the proportion of blood meals taken on humans and non-humans host. So, in the presence of odor-baited traps, the proportion of host-seeking mosquitoes that attempt to bite humans $Q_{\theta,t,h}$, is given by;

$Q_{\theta,t,h}=\left(\frac{N_{h}}{N_{h}+\alpha_{c}N_{c}+\alpha_{t}N_{t}}\right)=\left(\frac{N_{h}}{N_{h}+\alpha_{c}r_{c}N_{h}+\alpha_{t}r_{t}N_{h}}\right),$ (S1. 44)

Where, $\alpha_{c}$represents availability of cattle relative to humans and $r_{c}$represents ration of cattle to human.

Similarly, the proportion of host-seeking mosquitoes that bite cattle in presence of odor-baited traps, $Q_{\theta,t,c}$ (i.e., proportion of bites upon cattle prevented by traps) is expressed as follows

$Q_{\theta,t,c}=\left(\frac{\alpha_{c}r_{c}N_{h}}{N_{h}+\alpha_{c}r_{c}N_{h}+\alpha_{t}r_{t}N_{h}}\right),$ (S1. 45)

In the next sections, we will describe other interventions affecting a probability of a mosquito to feed successfully by considering interventions targeting mosquito while feeding on human both indoor and outdoor, and on non-human host. In addition, we will present the impact of interventions on mosquitoes while resting and searching for oviposition sites.

## **Targeting mosquitoes while attempting to feed on humans while indoors**

We describe the impact of in-house interventions, specifically, long-lasting insecticidal nets (ITNs), indoor residual spraying (IRS) and mosquito proofed housing (HM),

### Probability of a mosquito being repelled from an ITN or IRS treated house or due to Mosquito proofed housing (HM)

Let’s denote ITN coverage by $\chi_{LLIN}$, which we interpret as the proportion of people reported to be sleeping under long-lasting insecticidal nets, IRS coverage by $\chi_{IRS}$, which we interpret as the proportion of houses sprayed with insecticide, and mosquito proofed housing by $\chi_{HM}$, which we interpret as the proportion of houses which are mosquito proofed housing. Thus, the coverage parameters, $C_{LLIN}$, $C_{IRS}$, $C_{HM}$,$C_{LLIN,IRS}$, $C_{LLIN,HM}$,$C_{IRS,HM}$,$C_{LLIN,IRS,HM}$ and $C_{0}$represent coverage with ITN only, IRS only, HM only, ITN and IRS, ITN and HM, IRS and HM, ITN and IRS and HM, and with neither ITN or IRS or HM respectively. Assuming independent coverage of each intervention, then,

$c_{ITN}=\chi_{ITN}-\chi_{ITN}\chi_{IRS}-\chi_{ITN}\chi_{HM}+\chi_{ITN,IRS,HM}$ (S1. 46)

$c_{IRS}=\chi_{IRS}-\chi_{ITN}\chi_{IRS}-\chi_{IRS}\chi_{HM}+\chi_{ITN,IRS,HM}$ (S1. 47)

$c_{HM}=\chi_{HM}-\chi_{ITN}\chi_{HM}-\chi_{IRS}\chi_{HM}+\chi_{ITN,IRS,HM}$ (S1. 48)

$c_{ITN,IRS}=\chi_{ITN}\chi_{IRS}-\chi_{ITN,IRS,HM}$ (S1. 47)

$c_{ITN,HM}=\chi_{ITN}\chi_{HM}-\chi_{ITN,IRS,HM}$ (S1. 50)

$c_{IRS,HM}=\chi_{IRS}\chi_{HM}-\chi_{ITN,IRS,HM}$ (S1. 51)

$c_{ITN,IRS,HM}=\chi_{ITN}\chi_{IRS}\chi_{HM}$ (S1. 52)

$\begin{matrix} c_{0}=1-\chi_{ITN}-\chi_{IRS}-\chi_{HM}+\chi_{ITN}\chi_{IRS}+\chi_{ITN}\chi_{HM}+\chi_{IRS}\chi_{HM} \\ -\chi_{LLIN,IRS,HM} \end{matrix}$ (S1. 53)

**Fig C**. **Illustrations of the impact of indoor interventions.** It is assumed that repellency from mosquito proofed housing occurs prior to repellency from IRS and then repellency from IRS occurs prior to repellency from the ITNs. $r_{HM}$ or $d_{HM}$ , $r_{IRS}$ or $d_{IRS}$ and $r_{ITN}$ or $d_{ITN}$be a probability of mosquito being repelled or killed upon encountering ITNs, IRS, and HM respectively.

Now, let $r_{ITN}$, $r_{IRS}$ and $r_{HM}$ be a probability of mosquito being repelled upon encountering ITNs, IRS, and HM respectively, then, a probability of a mosquito being repelled from a house with any of the two or all three interventions is given by

$r_{ITN,IRS}=r_{IRS}+(1-r_{IRS})r_{ITN}$ (S1. 54)

$r_{ITN,HM}=r_{HM}+(1-r_{HM})r_{ITN}$ (S1. 55)

$r_{IRS,HM}=r_{HM}+(1-r_{HM})r_{IRS}$ (S1. 56)

$r_{ITN,IRS,HM}=r_{HM}+(1-r_{HM})r_{IRS}+(1-r_{HM})(1-r_{IRS})r_{ITN}$ (S1. 57)

If we let $\phi_{b}$, and $\phi_{i}$ with be a proportion of mosquito bites on a person while they are in bed and indoor respectively, then, a probability $(P)$ of a mosquito being repelled $(r)$ while attempting to feed indoor $(i)$ due to interventions $(\theta)$ ( i.e., ITNs and/or IRS and/or HM) is given by;

$p_{r,i,\theta}=Q_{\theta,t,h}[{C_{0}\phi_{i}r_{0}+C}_{HM}\phi_{i}r_{HM}+C_{ITN}\phi_{b}r_{ITN}+C_{IRS}\phi_{i}r_{IRS}+C_{ITN,HM}\left( \left( \phi_{i}-\phi_{b} \right)r_{HM}+\phi_{b}r_{ITN,HM} \right)+ C_{IRS,HM}\left( \phi_{i}r_{IRS,HM} \right)+C_{ITN,IRS}\left( \left( \phi_{i}-\phi_{b} \right)r_{IRS}+\phi_{b}r_{ITN,IRS} \right)+C_{ITN,IRS,HM}\left( {\left( \phi_{i}-\phi_{b} \right)r_{IRS,HM}+\phi}_{b}r_{ITN,IRS,HM} \right)]$ (S1. 58)

Where, $r_{0}$represents probability of a mosquito being repelled while attempting to feed indoor in the absence of interventions which is assumed to be 0.

### Probability that a mosquito successfully feeds and survives during a single feeding attempt in the presence of ITNs and/or IRS and/or HM

Recall that a mosquito successfully feeds by finding and feeding on a cattle or an unprotected human or a protected human. Thus, a probability $(P)$of successfully feeding $(f)$ and surviving during a single feeding attempt (i.e., only considering feeding on humans indoors ($(i)$) in presence of interventions $(\theta)$ (i.e., ITNs and/or IRS and/or HM) is given by:

$p_{f,i,\theta}=Q_{\theta,t,h} \left[ C_{HM}{((1-\phi}_{i} \right)+\phi_{i}s_{HM})+C_{ITN}((1-\phi_{b})+\phi_{b}s_{ITN}+C_{IRS}\left( \left( 1-\phi_{i} \right)+\phi_{i}s_{IRS} \right)+ C_{ITN,HM}{(\left( 1-\phi_{i} \right)+\left( \phi_{i}-\phi_{b} \right)s_{HM}+\phi}_{b}s_{ITN,HM})+ C_{IRS,HM}{(\left( 1-\phi_{i} \right)+\phi}_{i}s_{IRS,HM})+ C_{ITN,IRS}{(\left( 1-\phi_{i} \right)+\left( \phi_{i}-\phi_{b} \right)s_{IRS}+\phi}_{b}s_{ITN,IRS})+ C_{ITN,IRS,HM}{(\left( 1-\phi_{i} \right)+\left( \phi_{i}-\phi_{b} \right)s_{IRS,HM}+\phi}_{b}s_{ITN,IRS,HM})]$ (S1. 59)

Where, $\sigma_{0}$ is the probability of mosquitoes feeding successfully indoors in the absence of interventions assuming that the mosquito is not repelled.

Here, the probability of feeding and surviving upon encountering a house with ITNs is given by $S_{ITN}=\left( 1-r_{ITN} \right)\sigma_{ITN}$, the probability of feeding and surviving upon encountering an IRS-treated house is $S_{IRS}=\left( 1-r_{IRS} \right)\sigma_{IRS}$, and the probability of feeding and surviving upon encountering a mosquito proofed housing is given by $S_{HM}=\left( 1-r_{HM} \right)\sigma_{HM}$. Where, $\sigma_{ITN},$ $\sigma_{IRS},$ and $\sigma_{HM}$is the probability that mosquito survives and feeds after encountering a human in a house with an intervention (ITNs, IRS, and HM respectively), provided that it is not repelled. Thus, the probability of feeding and surviving upon encountering a house with either any of the two or all three interventions is given by;

$S_{ITN,HM}=S_{ITN}S_{HM},$ (S1. 60)

$S_{ITN,IRS}=S_{ITN}S_{IRS},$ (S1. 61)

$S_{IRS,HM}=S_{IRS}S_{HM},$ (S1. 62)

$S_{ITN,IRS,HM}=S_{ITN}S_{IRS}S_{HM}.$ (S1. 63)

Here, we have assumed that the mosquito must first avoid being repelled by mosquito proofed housing, then by IRS-treated house, and then must feed and survive mosquito proofed housing, the ITN and IRS treatment.

## **Targeting mosquitoes while attempting to feed on humans when outdoors**

Mosquito attempting to bite humans outdoor when are not protected by ITNs and/or IRS can potential be protected by spatial repellents and other personal protection measures (i.e., topical repellents and insecticide-treated clothing).

### Probability of a mosquito being repelled due to spatial repellent and/or other personal protection measures

Let’s denote spatial repellent coverage by $\chi_{SR}$ which we interpret as the proportion of people outdoor reported to be protected by spatial repellents, and personal protection measure coverage by $\chi_{PPM}$ which we interpret as the proportion of people reported to be protected by a personal protection measure. Thus, the coverage parameters, $C_{SR}$, $C_{PPM}$, $C_{SR,PPM}$ and $C_{PPM,SR,0}$represent coverage with spatial repellents only, personal protection measure only, spatial repellents and personal protection measure, and with neither spatial repellents or personal protection measure respectively. Assuming independent coverage of each intervention, then,

$C_{SR}=\chi_{SR}-\chi_{SR}\chi_{PPM}$ , (S1. 64)

$C_{PPM}=\chi_{PPM}-\chi_{PPM}\chi_{SR}$ , (S1. 65)

$C_{SR,PPM}=\chi_{SR}\chi_{PPM}$ , (S1. 66)

$C_{SR,PPM,0}=1-\chi_{SR}-\chi_{PPM}+\chi_{SR}\chi_{PPM}$ . (S1. 67)

Now, let $r_{SR}$ and $r_{PPM}$, be a probability of mosquito repeating a feeding attempt due to SR and PPM respectively, then, a probability of a mosquito being repelled due to SR and PPM is given by;

$r_{SR,PPM}=r_{SR}+(1-r_{SR})r_{PPM}$ (S1. 68)

Here, we have assumed that repellency from the spatial repellents occurs prior to repellency from a personal protection measure.

Now, $(1-\theta_{i})$ represents a proportion of mosquito bites on a person while they are outdoor, thus, a probability $(P)$of a mosquito being repelled $(r)$ while attempting to feed outdoor $(o)$ due to interventions $(\theta)$ (i.e., SR and PPM) is given by;

$p_{r,o,\theta}=Q_{\theta,t,h}(1-\theta_{i}(C_{SR}r_{SR}+C_{PPM}r_{PPM}+C_{SR,PPM}r_{SR,PPM})$ (S1. 69)

### Probability that a mosquito successfully feeds and survives during a single feeding attempt in the presence of spatial repellents and other personal protection measures

We can incorporate the impact of spatial repellents (SR) and other personal protection measures (PPM) by targeting the proportion of humans outdoor $Q_{h,0}\phi_{0}$. Thus, a probability $(P)$ of successfully feeding $(f)$ and surviving during a single feeding attempe (i.e., only considering feeding outdoor $(o)$) in presence of interventions $(\theta)$ (i.e., spatial repellents and personal protection measures) is given by,

$$p_{f,o,\theta}=Q_{\theta,t,h}(1-\theta_{i}(C_{SR}S_{SR}+C_{PPM}S_{PPM}+C_{SR,PPM}S_{SR,PPM}+C_{SR,PPM,0})$$

(S1. 70)

Where, the probability of feeding and surviving upon encountering an area with spatial repellents is given by $S_{SR}=(1-r_{SR})\sigma_{PPM}$, and the probability of feeding and surviving upon encountering a person with a personal protection measure is $S_{PPM}=(1-r_{PPM})\sigma_{PPM}$. Where, $\sigma_{SR}$ and $\sigma_{PPM}$represents a probability that a mosquito survives and feeds after encountering a human protected by spatial repellents and a personal protection measure respectively, provide that the mosquito is not repelled. Thus, the probability of feeding and surviving upon encountering both spatial repellents and personal protection measure is given by;

$S_{SR,PPM}=S_{SR}S_{PPM}$ (S1. 71)

Here, we have assumed that the mosquito must first avoid being repelled by the spatial repellents, and then must feed and survive both the spatial repellents and personal protection measure.

## **Targeting mosquitoes while attempting to feed on non-human host**

Several experiments have indicated that endocticides-treated cattle can reduce malaria transmission in humans. We explore the impact of systemic or topical insecticide applied to cattle in increasing mosquito mortality rate associated with insecticide contact during blood-feeding from cattle.

Let’s denote systemic applied insecticide coverage of cattle by $\chi_{cs}$ which we interpret as the proportion of cattle reported to be treated with systemic applied insecticide, and topical applied insecticide coverage of cattle by $\chi_{ct}$ which we interpret as the proportion of cattle reported to be treated with topical insecticide. Thus, the coverage parameters, $C_{cs}$, $C_{ct}$, $C_{cs,ct}$ and $C_{c,0}$represent coverage with systemic insecticide only, topical insecticide only, systemic and topical applied insecticide, and with neither systemic or topical insecticide respectively. Assuming independent coverage of each intervention, then,

$C_{cs}=\chi_{cs}-\chi_{cs}\chi_{ct}$ , (S1. 72)

$C_{ct}=\chi_{ct}-\chi_{cs}\chi_{ct}$ , (S1. 73)

$C_{cs,ct}=\chi_{cs}\chi_{ct}$ ,

$C_{c,0}=1-\chi_{cs}-\chi_{ct}+\chi_{cs}\chi_{ct}$ . (S1. 74)

Thus, a probability $(P)$of a mosquito being repelled $(r)$ while attempting to feed upon insecticide (topical) treated cattle $(c)$ is given by;

$P_{r,c,\theta}=Q_{\theta,t,c}[C_{c,ct}r_{ct}+C_{cs,ct}r_{ct}]$ (S1. 75)

Here, we assume that only topical applied insecticide on cattle can repel mosquitoes.

On the other hand, assuming that these interventions act independent of each other, then, a probability $(P)$ of successfully feeding $(f)$ a single feeding attempt on a cattle $(c)$ in presence of interventions $(\theta)$ (i.e., systemic or topical insecticide) is given by;

$P_{f,c,\theta}=Q_{\theta,t,c}[C_{c,0}+C_{cs}S_{cs}+C_{ct}S_{ct}+C_{cs,ct}S_{cs}S_{ct}]$ (S1. 76)

Here, $S_{cs}=(1-r_{cs})\sigma_{cs}$ and $S_{ct}=(1-r_{ct})\sigma_{ct}$ is the probability of a mosquito not being killed and feeding successfully on systemic and topical applied treated cattle respectively. Where, $\sigma_{cs}$ and $\sigma_{ct}$ represents the probability of mosquitoes feeding successfully outdoor in the presence of of systemic applied treated cattle and topical applied treated cattle respectively, provided that the mosquito is not repelled and $r_{cs}$ and $r_{ct}$ represents a probability of a mosquito being repelled after encountering a systemic and topical applied treated cattle respectively.

## Targeting mosquitoes while seeking resting sites, resting, and searching for oviposition sites

Mosquitoes can be targeted while searching for resting or oviposition sites, and resting after acquiring blood-meals by attractive toxic sugar baits (ATSB) and/or space spraying (SS) and/or using other traps (e.g., ovitraps) (OT) - these interventions can increase mosquito death rate $(\mu_{v,2})$.

Recall, if $\mu_{v,2}$ represents the natural death rate of an adult female mosquito while resting, finding larval habitat, and ovipositing, and then a mosquito rest, find larval habitat and oviposits during $t_{2}(0)$ days, then, the probability of surviving this process, $p_{2}$, in the absence of an intervention is given by $p_{2}=\exp(-\mu_{v,2}t_{2})$ respectively. Here, we can update $p_{2}$ to incorporate the impact of these interventions by considering their impact in increasing mosquito death rate $\mu_{v,2}$either alone or in combination.

Let’s denote ATSB coverage by$\chi_{ATBS}$, which we interpret as the proportion of vegetation sprayed with or outdoor baited stations provided with ATBS, and space spraying (SS) coverage by $\chi_{SS}$, which we interpret as the proportion of space sprayed with insecticide in a specific defined area,$\chi_{OT}$, which we interpret as the proportion of other traps (OT) capable of kiITNg resting and/or ovipositing mosquitoes. Thus, the coverage parameters, $C_{ATSB}$, $C_{SS}$, $C_{OT}$,$C_{ATSB,SS}$, $C_{ATSB,OT}$,$C_{SS,OT}$,$C_{ATSB,SS,OT}$ and $C_{ATSB,SS,OT,0}$represent coverage with ATSB only, SS only, OT only, ATSB and SS, ATSB and OT, SS and OT, ATSB and SS and OT, and with neither ATSB or SS or OT respectively. Assuming independent coverage of each intervention, then,

$c_{ATSB}=\chi_{ATSB}-\chi_{ATSB}\chi_{SS}-\chi_{ATSB}\chi_{OT}+\chi_{ATSB,SS,OT}$ (S1. 77)

$c_{SS}=\chi_{SS}-\chi_{ATSB}\chi_{SS}-\chi_{SS}\chi_{OT}+\chi_{ATSB,SS,OT}$ (S1. 78)

$c_{OT}=\chi_{OT}-\chi_{ATSB}\chi_{OT}-\chi_{SS}\chi_{OT}+\chi_{ATSB,SS,OT}$ (S1. 79)

$c_{ATSB,SS}=\chi_{ATSB}\chi_{SS}-\chi_{ATSB,SS,OT}$ (S1. 80)

$c_{ATSB,OT}=\chi_{ATSB}\chi_{OT}-\chi_{ATSB,SS,OT}$ (S1. 81)

$c_{SS,OT}=\chi_{SS}\chi_{OT}-\chi_{ATSB,SS,OT}$ (S1. 82)

$C_{ATSB,SS,OT}=\chi_{ATSB}\chi_{SS}\chi_{OT}$ (S1. 83)

$\begin{matrix} c_{ATSB.SS,OT,0}=1-\chi_{ATSB}-\chi_{SS}-\chi_{OT}+\chi_{ATSB}\chi_{SS}+\chi_{ATSB}\chi_{OT}+\chi_{SS}\chi_{OT} \\ -\chi_{ATSB,SS,OT} \end{matrix}$ (S1. 84)

Where, the coverage of ovitraps is adjusted by its effectiveness so $\chi_{OT}=\varepsilon_{OT}\chi_{OT}$. Ovitraps can also reduce the number of eggs laid by a female mosquito – this impact is incorporated in Equation 90. If we let $\zeta_{ATSB}$, $\zeta_{SS}$, and $\zeta_{OT}$ be the factor allowing for increased $\mu_{v,2}$ in presence of ATSB, SS, and OT respectively, then the death rate due ATBS and/or SS and/or OT is be given by:

$\mu_{v,2,\theta}= \mu_{v,2}\left[\begin{matrix} C_{ATSB}\zeta_{ATSB}+C_{SS}\zeta_{SS}+C_{OT}\zeta_{OT}+C_{ATSB,SS}\zeta_{ATSB}\zeta_{SS}+C_{ATSB,OT}\zeta_{ATSB}\zeta_{OT}+C_{SS,OT}\zeta_{SS}\zeta_{OT}+ \\ C_{ATSB,SS,OT}\zeta_{ATSB}\zeta_{SS}\zeta_{OT}+C_{ATSB,SS,OT,0} \end{matrix}\right]$ (S1. 85)

Thus, the probability of surviving host-seeking process in the presence of these interventions can be given by:

$P_{2,\theta}=e^{-\mu_{V,2,\theta}t_{2}}$ (S1. 86)

The Impact of combined interventions on mosquito population parameters

At this stage, we can now present the impact of described interventions on the important mosquito population parameters.

### Calculating the length of gonotrophic cycle in the presence of intervention

First, we compute the total probability of a mosquito to reset and begin a new search $p_{r}$ by combining the probabilities of being repelled while attempting to feed upon human indoor (Equation S1. 58) and outdoor (Equation S1. 69) and upon cattle (Equation S1. 75) as follows:

$P_{r,\theta}=p_{r,i,\theta}+p_{r,o,\theta}+p_{r,c,\theta}$ (S1. 87)

Then, the length of gonotrophic cycle in presence of interventions $(f_{\theta})$ is given by

$f_{\theta}=\frac{t_{1}(0)}{1-P_{r,\theta}}+t_{2}$ (S1. 88)

### Calculating the probability of surviving one day

First, we compute the total probability of surviving feeding by combining the probabilities of feeding on humans indoor (Equation S1. 59 ) and outdoor (Equation S1. 70) and non-human host (Equation S1. 76) as follows;

$P_{f,\theta}=p_{f,i,\theta}+p_{f,o,\theta}+p_{f,c,\theta}$ (S1. 89)

Then, the probability of surviving feeding period, $P_{1,\theta}$, in presence of an intervention (updating equation S1. 43) is given by

$p_{1,\theta}=e^{-\mu_{V,1,\theta}\left(\frac{t_{1}}{1-P_{r,\theta}}\right)}$ (S1. 90)

Thus, probability of surviving one day in presence of interventions is

$P_{\theta}=\left(p_{1,\theta}p_{f,\theta}p_{2,\theta}\right)^{1/f_{\theta}}$ (S1. 91)

### Computing female mosquito death rate in presence of interventions

Mosquito daily death rate due to an intervention can be computed as follows;

$\overline{\mu_{v,\theta}}=-\log(P_{\theta})$ (S1. 92)

### Calculating the number of eggs laid per day per a female mosquito in the presence of interventions

Given $\varepsilon$ is the number of viable eggs that a female mosquito lays per oviposition cycle, the rate at which female mosquitoes oviposit eggs, $\beta_{\theta}$, in presence of interventions is given by,

$\beta_{\theta}=\frac{\varepsilon\overline{\mu_{v}}_{,\theta}}{e^{\overline{\mu_{V}}/f(\theta)}-1}(1-\varepsilon_{OT}\chi_{OT})$ (S1. 93)

Where, the term $(1-\varepsilon_{OT}\chi_{OT})$captures the impact of ovitraps in reducing the number of eggs.

Expected Model Outputs

The model can be used to produce a number of interesting outputs as desired by the user. We present mathematical expression of each possible model output. The main results will be presented based on the optimal intervention packages that can be used to reduce annual entomological inoculation rates (EIR) to levels below 1 – required to interrupt malaria transmission.

## Reducing mosquito density in presence of interventions

Mosquito density in presence of interventions can be computed by dividing mosquito population (M) at time (t) with total population of humans (H) as follows:

$m_{\theta}=\frac{V(t)}{H}$ (S1. 94)

## Reducing the proportion of blood-meals obtained from human in presence of interventions

The proportion of blood meals that are taken on humans in the present of intervention can be computed from Equations 28, 33, and 38 as follows:

$Q_{h,\theta}=\frac{p_{f,i,\theta}+p_{f,o,\theta}}{P_{f,\theta}}$ (S1. 95)

## Reducing human biting rate per mosquito in presence of interventions

The human biting rate per mosquito in presence of interventions can be given by;

$a_{\theta}=\frac{Q_{h,\theta}}{f_{\theta}}$ (S1. 96)

## Reducing vectorial capacity in presence of interventions

The vectorial capacity can be computed as follows:

$VC_{\theta}=\frac{m_{\theta}\left(\frac{Q_{\theta}}{f_{\theta}}\right)^{2}e^{-\overline{\mu}_{V}\tau}}{\overline{\mu}_{V}}$ (S1. 97)

Where,$\tau$ is the average incubation period in days - a delay before mosquito become infectious after being infected.

## Reducing entomological inoculation rate in presence of interventions

Malaria transmission intensity is often expressed in terms of the entomologic inoculation rate (EIR) which is a direct, field- measurable indicator of human exposure to bites of mosquitoes infected with transmissible sporozoite stage malaria parasites. EIR in presence of interventions can be computed as follows:

$EIR_{\theta}=\frac{I_{V}(t)a_{\theta}}{H}$ (S1. 98)

Here, $I_{V}(t)$ assumed to be computed daily resulting to daily EIR – so yearly EIR can be computed by multiplying Equation 95 by 365.

## Estimating basic reproduction number in presence of interventions

The basic reproduction number represents the number of new infections one case introduces to a susceptible population on average over the course of its infectious period. R_0_ is an important indicator because it helps specify the disease condition (i.e., if R_0_ < 1 the infection will disappear in the long run; R_0_ = 1 is the endemic state; and if R_0_ > 1 the infection will be able to spread in the population. The higher R_0_, the harder it is to control the infection. R_0_ can be estimated directly as,

$R_{0,\theta}=\left(\frac{V}{H}\right)a_{\theta}^{2}b_{v}\frac{b_{h}e^{-\overline{\mu}_{V}\tau}}{\overline{\mu_{v,\theta}}\nu}$ (S1. 99)

Where, $\nu$ is recovery rate - all other terms as previously defined.

## Additional Results

In this subsection, we present additional results not presented in the main manuscript focusing on *An. funestus*

### Sensitivity analysis

In addition to sensitivity analysis results presented in the main document for *An. gambiae s.s.* and *An. arabiensis,* we also performed sensitivity analysis to determine which parameters have great influence on the model output i.e., entomological inoculation rate, based on the impact of 50% coverage of ITNs, attractive toxic sugar baits (ATSB), topical (ECT) endectocide-treated cattle, mosquito-proofed housing (HOU), larviciding (LAR) and personal protection measure (PPM) on *An. funestus*. As shown in Fig D, a factor allowing for increased mosquito death rate due to ATSB ( *fATST*), probability of mosquito being repelled upon encountering a mosquito proofed housing (*rHOU*) and insecticide treated nets (*rITN*), a probability of feeding and surviving upon encountering a mosquito proofed housing (*sHOU*) and a personal protection measure (*sPPM*), mosquito death rate while searching for blood (*muV*), a proportion of mosquito bites on a person while they are indoor (*phiI*), and a probability of - all have the most influence on the model output for *An. funestus*.

**Fig D: Sensitivity analysis results for *An. funestus*:** Latin Hypercube Sampling/Partial Rank Correlation Coefficient (LHS/PRCC) sensitivity analysis approach was implemented to perform sensitivity analysis based on the impact of selected vector control tools in reducing entomological inoculation rate for *An. funestus****.***

### Evaluating the impact of combining ITNs at 50% and 80% coverage with additional tools to control An. funestus

In addition to the results presented for *Anopheles gambiae s.s*. and *An. arabiensis* in the main document - the model was used to investigate additional interventions to ITNs at 50% and 80% coverage respectively that would be successful in interrupting malaria transmission for vector populations predominated by *An. funestus* in all the three transmission settings (Fig E).

**Fig E: Evaluating the impact of combining ITNs at 50% and 80% coverage with additional tools against *An. funestus*:** The tools selected in this example are attractive toxic sugar baits (ATSB), topical (ECT) endectocide-treated cattle, mosquito-proofed housing (HOU), larviciding (LAR) and personal protection measure (e.g. insecticide-treated clothing) (PPM). Adding one or two tools to ITNs at 50% coverage might be sufficient to interrupt transmission in low transmission settings (**A**); but in most cases not sufficient in moderate (**C**) and high transmission (**E**) settings. Scaling up ITNs to 80% coverage and adding another tool with 50% coverage might be sufficient to interrupt transmission in low (**B**) transmission but not necessarily in moderate (**D**) and high (**F**) transmission settings

As shown in Figure E, adding one and even a second intervention at 50% coverage might not be sufficient to interrupt high (panel **E**) or moderate transmission (panel **C**). On the other hand, adding mosquito proofed housing (HOU) at 50% might be sufficient to interrupt low (panel **A**) transmission against *An. funestus.* By scaling up ITNs to 80% coverage and maintaining additional tools at 50% coverage, then adding one of the selected intervention should be sufficient to interrupt transmission against *An. funestus* in areas with low (panel **B**) malaria transmission.

# References

1. White MT, Griffin JT, Churcher TS, Ferguson NM, Basáñez M-G, Ghani AC. Modelling the impact of vector control interventions on Anopheles gambiae population dynamics. Parasit Vectors. 2011;4(1):1.

2. Griffin JT, Hollingsworth TD, Okell LC, Churcher TS, White M, Hinsley W, et al. Reducing Plasmodium falciparum malaria transmission in Africa: a model-based evaluation of intervention strategies. PLoS Med. 2010;7(8):e1000324.
