# Supplementary material for: Attacking the mosquito on multiple fronts: Insights from the Vector Control Optimization Model (VCOM) for malaria elimination
Source: PLoS One. 2017 Dec 1;12(12):e0187680. doi: 10.1371/journal.pone.0187680 (PMC5711017; doi:10.1371/journal.pone.0187680)
Supplement: S2 Appendix — (DOCX) [file pone.0187680.s011.docx]

**Attacking the mosquito on multiple fronts: Insights from the Vector Control Optimization Model (VCOM) for malaria elimination**

Samson S. Kiware­^1,2^, Nakul Chitnis^3,4^, Allison Tatarsky^5^, Sean Wu^6^, Héctor Manuel Sánchez Castellanos^6,7^, Roly Gosling^5^, David Smith^8^, John Marshall^6^

1. *Biomedical and Environmental Thematic Group, Ifakara Health Institute, Morogoro,Tanzania*
2. *Mathematics, Statistics, and Computer Science Department, Marquette University, Milwaukee, WI, USA*
3. *Department of Epidemiology and Public Health, Swiss Tropical and Public Health Institute, Basel, Switzerland*
4. *University of Basel, 4003 Basel, Switzerland*
5. *Malaria Elimination Initiative, Global Health Group, University of California, San Francisco, USA*
6. *Divisions of Biostatistics and Epidemiology, University of California, Berkeley*
7. *School of Medicine, Tecnologico de Monterrey, Atizapan de Zaragoza, Estado de Mexico, 64849,*
8. *Mexico Department of Global Health, University of Washington, Seattle, USA*

**S2 Appendix**

**Parameter Values**

This document provides the parameter values for the vector species considered (i.e. *Anopheles gambiae*, *An. arabiensis* and *An. funestus*) and human components of the model and intervention parameter values, obtained from detailed literature review. Intervention parameters are assumed to remain constant for the duration of the control program.

**Table A:** Parameter values for the vector and human components of the model considered the same between the three mosquito species

**Table B:** Basic parameter estimates for the vector ecology and control model which vary between mosquito species.

**Table C:** Parameter for the effectiveness of all interventions considered.

**REFERENCES:**

1. Smith D, Dushoff J, Snow R, Hay S. The entomological inoculation rate and Plasmodium falciparum infection in African children. Nature. 2005;438(7067):492-5.

2. Costantini C, LI SG, Torre AD, Sagnon NF, Coluzzi M, Taylor CE. Density, survival and dispersal of Anopheles gambiae complex mosquitoes in a West African Sudan savanna village. Med Vet Entomol. 1996;10(3):203-19.

3. Gillies M. Observations on nulliparous and parous rates in some common East African mosquitoes. Ann Trop Med Parasitol. 1963;57(4):435-42.

4. Gu W, Mbogo CM, Githure JI, Regens JL, Killeen GF, Swalm CM, et al. Low recovery rates stabilize malaria endemicity in areas of low transmission in coastal Kenya. Acta Trop. 2003;86(1):71-81.

5. Killeen GF, McKenzie FE, Foy BD, Schieffelin C, Billingsley PF, Beier JC. A simplified model for predicting malaria entomologic inoculation rates based on entomologic and parasitologic parameters relevant to control. Am J Trop Med Hyg. 2000;62(5):535-44.

6. Beier JC, Oster CN, Onyango FK, Bales JD, Sherwood JA, Perkins PV, et al. Plasmodium falciparum incidence relative to entomologic inoculation rates at a site proposed for testing malaria vaccines in western Kenya. Am J Trop Med Hyg. 1994;50(5):529-36.

7. Dietz K, Molineaux L, Thomas A. A malaria model tested in the African savannah. Bulletin of the World Health Organization. 1974;50(3-4):347.

8. Rickman LS, Jones TR, Long GW, Paparello S, Schneider I, Paul CF, et al. Plasmodium falciparum-infected Anopheles stephensi inconsistently transmit malaria to humans. Am J Trop Med Hyg. 1990;43(5):441.

9. Garrett-Jones C, Shidrawi G. Malaria vectorial capacity of a population of Anopheles gambiae: an exercise in epidemiological entomology. Bulletin of the World Health Organization. 1969;40(4):531.

10. Charlwood J, Smith T, Billingsley P, Takken W, Lyimo E, Meuwissen J. Survival and infection probabilities of anthropophagic anophelines from an area of high prevalence of Plasmodium falciparum in humans. B Entomol Res. 1997;87(05):445-53.

11. Service M. Studies on sampling larval populations of the Anopheles gambiae complex. Bulletin of the World Health Organization. 1971;45(2):169.

12. Bayoh M, Lindsay S. Effect of temperature on the development of the aquatic stages of Anopheles gambiae sensu stricto (Diptera: Culicidae). Bull Entomol Res. 2003;93(05):375-81.

13. Killeen GF, McKenzie FE, Foy BD, Schieffelin C, Billingsley PF, Beier JC. A simplified model for predicting malaria entomologic inoculation rates based on entomologic and parasitologic parameters relevant to control. Am J Trop Med Hyg 2000;62(5):535-44.

14. Killeen GF, Smith TA. Exploring the contributions of bed nets, cattle, insecticides and excitorepellency to malaria control: a deterministic model of mosquito host-seeking behaviour and mortality. Trans R Soc Trop Med Hyg. 2007;101(9):867-80.

15. Tirados I, Costantini C, Gibson G, Torr SJ. Blood‐feeding behaviour of the malarial mosquito Anopheles arabiensis: implications for vector control. Med Vet Entomol. 2006;20(4):425-37.

16. Garrett-Jones C, Grab B. The assessment of insecticidal impact on the malaria mosquito's vectorial capacity, from data on the proportion of parous females. Bulletin of the World Health Organization. 1964;31(1):71.

17. Githeko A, Mbogo C, Atieli F. Resting behaviour, ecology and genetics of malaria vectors in large scale agricultural areas of Western Kenya. Parassitologia. 1996;38(3):481-9.

18. Killeen GF, Kihonda J, Lyimo E, Oketch FR, Kotas ME, Mathenge E, et al. Quantifying behavioural interactions between humans and mosquitoes: evaluating the protective efficacy of insecticidal nets against malaria transmission in rural Tanzania. BMC Infect Dis. 2006;6(1):161.

19. Mathenge EM, Gimnig JE, Kolczak M, Ombok M, Irungu LW, Hawley WA. Effect of permethrin-impregnated nets on exiting behavior, blood feeding success, and time of feeding of malaria mosquitoes (Diptera: Culicidae) in western Kenya. J Med Entomol. 2001;38(4):531-6.

20. Curtis C, Myamba J, Wilkes T. Comparison of different insecticides and fabrics for anti‐mosquito bednets and curtains. Med Vet Entomol. 1996;10(1):1-11.

21. Smith A, Webley D. A veranda-trap hut for studying the house-frequenting habits of mosquitoes and for assessing insecticides. III. The effect of DDT on behaviour and mortality. Bull Entomol Res. 1969;59(01):33-46.

22. Djènontin A, Bio-Bangana S, Moiroux N, Henry M-C, Bousari O, Chabi J, et al. Culicidae diversity, malaria transmission and insecticide resistance alleles in malaria vectors in Ouidah-Kpomasse-Tori district from Benin (West Africa): A pre-intervention study. Parasit & Vectors. 2010;3(1):83.

23. Ogoma SB, Ngonyani H, Simfukwe ET, Mseka A, Moore J, Maia MF, et al. The mode of action of spatial repellents and their impact on vectorial capacity of Anopheles gambiae sensu stricto. PloS one. 2014;9(12):e110433.

24. Kimani EW, Vulule JM, Kuria IW, Mugisha F. Use of insecticide-treated clothes for personal protection against malaria: a community trial. Mal J. 2006;5(1):63.

25. Fritz M, Siegert P, Walker E, Bayoh M, Vulule J, Miller J. Toxicity of bloodmeals from ivermectin-treated cattle to Anopheles gambiae sl. Ann Trop Med Parasitol. 2009;103(6):539-47.

26. Marshall JM, White MT, Ghani AC, Schlein Y, Muller GC, Beier JC. Quantifying the mosquito’s sweet tooth: modelling the effectiveness of attractive toxic sugar baits (ATSB) for malaria vector control. Malaria journal. 2013;12(1):1.

27. Yakob L, Yan G. Modeling the effects of integrating larval habitat source reduction and insecticide treated nets for malaria control. PLoS One. 2009;4(9):e6921.

28. Organization WH. World Malaria Report. Geneva. 2011.

29. Kamareddine L. The biological control of the malaria vector. Toxins. 2012;4(9):748-67.
