# Supplementary material for: Attacking the mosquito on multiple fronts: Insights from the Vector Control Optimization Model (VCOM) for malaria elimination
Source: PLoS One. 2017 Dec 1;12(12):e0187680. doi: 10.1371/journal.pone.0187680 (PMC5711017; doi:10.1371/journal.pone.0187680)
Supplement: S3 Appendix — (DOCX) [file pone.0187680.s012.docx]

**Attacking the mosquito on multiple fronts: Insights from the Vector Control Optimization Model (VCOM) for malaria elimination**

Samson S. Kiware­^1,2^, Nakul Chitnis^3,4^, Allison Tatarsky^5^, Sean Wu^6^, Héctor Manuel Sánchez Castellanos^6,7^, Roly Gosling^5^, David Smith^8^, John Marshall^6^

1. *Biomedical and Environmental Thematic Group, Ifakara Health Institute, Morogoro,Tanzania*
2. *Mathematics, Statistics, and Computer Science Department, Marquette University, Milwaukee, WI, USA*
3. *Department of Epidemiology and Public Health, Swiss Tropical and Public Health Institute, Basel, Switzerland*
4. *University of Basel, 4003 Basel, Switzerland*
5. *Malaria Elimination Initiative, Global Health Group, University of California, San Francisco, USA*
6. *Divisions of Biostatistics and Epidemiology, University of California, Berkeley*
7. *School of Medicine, Tecnologico de Monterrey, Atizapan de Zaragoza, Estado de Mexico, 64849,*
8. *Mexico Department of Global Health, University of Washington, Seattle, USA*

# S3 Appendix

# Graphic User Interface

VCOM can be found as a stand-alone application that runs in any modern browser for users to perform their own model runs. The software gives users complete control over the model’s parameters to replicate all results presented herein, as well as to investigate settings specific to their own needs. The functionalities of VCOM are self-explanatory and the GUI itself is developed in such a way that it is easier to use. VCOM can be found here: <http://skiware.github.io/VCOM/>

The initial splash page allows users to choose either the “Simple GUI” or “Advanced GUI”. While both link to the same model equations, they differ in the level of control the user has over parameter specifications. After navigating to the simple or advanced menu, the user will be presented with the GUI as shown below.

## Simple GUI

The simple interface is designed for users who may not necessarily possess a background in either entomology or epidemiology to explore the effects of different combinations of vector control interventions on various metrics of epidemiological and ecological importance (see Figure F). The simple interface allows users to select which mosquito species the model will be parameterized to simulate (currently, *An. Gambiae*, *An. Funestus* or *An. Arabiensis*), the baseline entomological inoculation rate (EIR), *Q­*_0_ (proportion of bloodmeals taken on humans), and how long to run the simulation for.

The user also has control over basic parameters related to interventions. For each of the 13 interventions included, users can select the percentage level of coverage and the time at which the intervention is “turned on”.

The model will output a line plot of change in vector densities over time and a histogram which displays the mean population size in each compartment of the model.

**Fig F. Illustrations of VCOM’s Simple Graphic User Interface.** The simple interface allows users to select which mosquito species the model will be parameterized to simulate, the baseline entomological inoculation rate (EIR), select the percentage level of coverage for desired interventions, and how long to run the simulation for.

## Advanced GUI

The advanced interface is designed for users who may themselves be epidemiologists, entomologists, or anyone with a good understanding of mosquito bionomic parameters and vector control interventions. It allows near-complete control of all model parameters through a downloadable .xml file in which users input desired parameter values. This .xml file is then uploaded to the advanced interface and the model is run. The advanced interface allows the user to download detailed numerical results from the ODE integrators, as well as derived numerical parameters (such as EIR, R­_0_, and VC).
